# Identifying the deceiver: the non-neoplastic mimickers of genital system neoplasms

**DOI:** 10.1186/s13244-021-01046-x

**Published:** 2021-07-07

**Authors:** Omer Onder, Ali Devrim Karaosmanoglu, Jessica Kraeft, Aycan Uysal, Musturay Karcaaltincaba, Deniz Akata, Mustafa Nasuh Ozmen, Peter F. Hahn

**Affiliations:** 1 Department of Radiology, Hacettepe University School of Medicine, Ankara, 06100 Turkey; 2 Department of Radiology, University of Colorado School of Medicine, Aurora, CO 80045 USA; 3 Department of Radiology, Gulhane Training and Research Hospital, Ankara, 06010 Turkey; 4 Department of Radiology, Massachusetts General Hospital, Harvard Medical School, Boston, MA 02114 USA

**Keywords:** Genital system, Neoplasm, Infection, Inflammation, Mimicker

## Abstract

Tumors of the genital system are common and imaging is of crucial importance for their detection and diagnosis. Several non-neoplastic diseases may mimic these tumors and differential diagnosis may be difficult in certain cases. Misdiagnosing non-neoplastic diseases as tumor may prompt unnecessary medical treatment or surgical interventions. In this article, we aimed to present the imaging characteristics of non-neoplastic diseases of the male and female genital systems that may mimic neoplastic processes. Increasing awareness of the imaging specialists to these entities may have a severe positive impact on the management of these patients.

## Key points

- Genital organs are commonly affected by neoplastic and non-neoplastic diseases in both sexes and imaging plays a crucial role in differential diagnosis.
- Several infectious and inflammatory disorders may mimic cancer both clinically and radiologically in both sexes.
- Familiarity with the imaging findings of these different non-neoplastic urogenital disorders may be very helpful for the correct diagnosis.

## Introduction

Genital organs are commonly affected by neoplastic diseases in both sexes. In these patients, imaging studies play a fundamental role in the detection, differential diagnosis, staging, and follow-up. It should be noted that several non-neoplastic diseases may also present with similar findings and differentiation from neoplastic diseases may be difficult in some patients. Clinical and laboratory findings may provide additional information in selected cases. Histopathologic confirmation will often be needed for treatment guidance in these patients. The major goal of this paper is to describe various imaging aspects of several non-neoplastic conditions affecting the male and female genital organs.

## Male genital tract

The male genital tract may harbor various non-neoplastic diseases that can mimic malignancy. A comprehensive summary of clinical and radiological findings of tumor mimickers in the male genital tract can be found in Tables 1 and 2.

**Table 1:** Clinical and radiological findings of tumor mimickers in the male genital tract (part-1)

Disease	Imaging findings	Auxiliary clinical information
Chronic bacterial prostatitis	Linear, less mass-forming lesions with ill-defined borders. Lobar distribution or diffuse involvement of the peripheral zone. Low T2 signal. Tends to have less diffusion restriction than neoplasms. Early arterial enhancement may be seen	Systemic symptoms are not expected. Fluctuant PSA elevation, decrease in PSA levels after antibiotic treatment. In challenging cases, targeted prostate biopsy may be needed
Prostatic tuberculosis	Nodular form: T1-isointense, T2-hypointense multiple nodules of varying sizes with restricted diffusion. Extraprostatic extension is mostly not expected Diffuse form: More common form. Restricted diffusion may be seen	History of systemic tuberculosis or intravesical BCG instillation Targeted prostate biopsy is required for definitive diagnosis
Prostatic involvement of granulomatous with polyangiitis	Low T2 signal. Low ADC values. Can simulate prostate cancer and prostatic abscesses	Medical history for granulomatous polyangiitis. Histopathological examination is needed
IgG4-related prostatitis	There is not much radiological information in the literature due to the rarity of the disease. FDG-avid lesions	Rare. History of IgG4-RD and accompanying organ involvements may be clue
Mumps orchitis	Acute phase: Enlarged testis with increased vascularity. Scrotal wall thickening and epididymal involvement are common Chronic phase: Atrophy with parenchymal heterogeneity	Seen in pubertal and postpubertal period. Infertility. Medical history for mumps Negative serum tumor markers may be helpful
Tuberculous orchitis	Generally coexists with epididymal involvement (heterogeneously enlarged epididymis) US: Diffusely enlarged homogeneous/heterogeneous hypoechoic testis or multiple small-sized hypoechoic nodules. Scrotal abscesses, sinus tracts, skin thickening, complex hydrocele, calcification MR: Mostly seen as T2 hypointense, T1 hyperintense lesions with variable enhancement	Isolated testicular involvement is rare. History of tuberculosis or immunosuppression may be suggestive. Typical clinical findings of acute infection are not expected. Microbiological and histopathological examinations are required for definitive diagnosis
Xanthogranulomatous orchitis	Large, heterogeneous masses without apparent vascularity. Parenchymal calcifications, cystic changes and epididymal involvement may also accompany	Very rare. Increased coexistence with diabetes. Urine cultures may grow *E. coli* and *P. aeruginosa*
Testicular malakoplakia	Unilateral testicular enlargement, heterogeneous mass with cystic areas and intratesticular abscesses may be seen	Rare inflammatory disease. May present with symptoms related to epididymoorchitis. Histopathological examination is required for definitive diagnosis
Testicular abscess	US: Hypoechoic lesions with ill-defined “shaggy” walls. Increased perilesional and absent intralesional vascularity. May appear solid-like, and mimic testicular neoplasms MRI: Contrast enhancing wall and intralesional non-enhancing component	May be secondary to the bacterial epididymo-orchitis, pre-exiting hematomas or infarct areas. Clinical history, symptoms and acute phase reactants may be helpful Percutaneous FNAB or even surgery may be required
Testicular necrotising vasculitis	US: Heterogeneous hypoechoic focal parenchymal lesion with variable internal vascularity Multifocal involvement may also be seen	History of polyarteritis nodosa (as the most common), granulomatosis with polyangiitis, Churg-Strauss syndrome, giant cell arteritis, Henoch-Schonlein purpura
Testicular involvement in IgG4-RD	There is not much information in the literature due to the rarity of the disease. In one case report, hypoechoic focal mass is defined	Extremely rare. History of IgG4-RD and accompanying organ involvements
Testicular sarcoidosis	Multiple hypoechoic testicular lesions with/without epididymal involvement. May also present with solitary mass. Internal vascularity is not expected on color Doppler US	Uncommon. Patient demographics. History of sarcoidosis. Elevated serum ACE levels may be helpful

**Table 2:** Clinical and radiological findings of tumor mimickers in the male genital tract (part-2)

Disease	Imaging findings	Auxiliary clinical information
Testicular hematoma	Acute phase (US): Iso-hyperechoic mass with no apparent internal vascularity Resolution phase (US): Hypoechoic lesion with gradual decrease in size Single or multiple lesions can be seen. Concomitant imaging findings of scrotal trauma such as hematocele MRI: T1 hyperintensity (subacute phase), T2 hypointensity (chronic phase), no internal enhancement	History of trauma is suggestive. Resolution during follow-up is expected. May complicate with testicular necrosis, compression atrophy or secondary infections. Incidental diagnosis of testicular tumors after scrotal trauma should also be kept in mind
Segmental testicular infarction	US: Solid-appearing wedge-shaped heterogeneous lesion without apparent vascularity MRI: Well-defined heterogeneous parenchymal areas with absence of enhancement. Hemorrhagic changes may be seen as T1 hyperintense areas. Rim enhancement may be observed	Testicular pain. Young adults. Underlying causes may be infection, trauma, vasculitis, or hematologic diseases. Follow-up imaging is required to rule out malignancy
Leydig’s cell hyperplasia	US: Small, multifocal, frequently bilateral parenchymal nodules with internal vascularity and variable echogenicity MRI: T2 hypointense nodules with avid contrast enhancement	Rare. Mostly seen in asymptomatic patients with normal serum tumor markers. May coexist with Klinefelter syndrome
Testicular adrenal rests	US: Bilateral, multiple, well-defined hypoechoic nodular lesions with no apparent mass effect located in the mediastinum testis. Tend to appear as heterogeneous hyperechoic masses as they get larger. Internal vascularity is variable. Posterior acousting shadowing may be observed MR: T1 iso-hyperintense, T2-hypointense lesions with avid enhancement	History of congenital adrenal hyperplasia or Cushing’s syndrome. May cause infertility. Regression after glucocorticoid therapy can be seen
Tubular ectasia of rete testis	US: Multiple cystic-tubular anechoic structures replacing the mediastinum testis. Internal vascularity, calcification, presence of solid component are not expected. Bilateral involvement is common MRI: T2-hyperintense cystic structure with no internal enhancement	Over the age of 45. History of previous scrotal surgery and vasectomy. It should be kept in mind that testicular tumors may also cause post-obstructive rete testis dilation
Cystic dysplasia of rete testis	Unilateral, multicystic lesion compressing the surrounding parenchyma in the mediastinum testis It may be useful to control ipsilateral renal anomalies such as renal agenesis or multicystic dysplastic kidney	Seen in the pediatric age group. Unilateral painless scrotal swelling. May coexist with ipsilateral renal anomalies. Testicular compression atrophy may develop Negative serum tumor markers
Paratesticular inflammatory pseudotumor	US: Paratesticular lesions with variable echogenicity and mild-to-moderate internal vascularity. Posterior acoustic shadowing and intralesional calcifications may be seen MRI: T1 and T2 hypointense lesions with gradual contrast enhancement	Uncommon. May be related to previous surgery, trauma, infection or inflammation. Could be secondary to the IgG4-RD
Paratesticular tuberculoma	US: Well-circumscribed hypoechoic lesions with calcifications and mild internal vascularity. Extension to the scrotal wall or testicular parenchyma may be seen MRI: T1-hyperintense and T2-hypointense paratesticular lesions	Rare. History of immunosuppression or intravesical BCG instillation. Histopathological examination is required for definitive diagnosis
Polyorchidism	US: Solid nodular lesion with similar echogenicity and echotexture to the normal testes MRI: Homogeneous solid lesions with intermediate T1 and high T2 signal intensity similar to testes	Rare congenital anomaly. More common on the-left side. Extra testis is smaller than normal testes, and mostly located within the scrotum adjacent to the lower pole
Splenogonadal fusion	Continuous form: Long parenchymal or fibrous cord extending from the spleen to the testis Discontinuous form: Homogeneous solid iso-hypoechoic lesion with internal vascularity In case of suspicion, Tc99m-sulfur colloid scintigraphy can help detection and prevent orchiectomy	Rare congenital anomaly. More common on the left-side. Commonly associated with inguinal hernia and cryptorchidism

## Prostate

Prostate cancer is an extremely common malignancy. Multiparametric prostate MRI has been applied successfully for diagnosis and lesion localization. Typical MRI features of prostate cancer include low signal intensity on T2-weighted (T2W) images with corresponding diffusion restriction. Early phase enhancement is typical on dynamic contrast-enhanced (DCE) T1-weighted (T1W) images, which is another helpful feature for differential diagnosis. Several mimics of cancer should be considered during the interpretation of these studies [1].

### Bacterial prostatitis

Bacterial prostatitis may manifest in acute or chronic forms. Acute bacterial prostatitis is mostly seen in young patients. The disease presents typically with a combination of lower urinary tract symptoms and systemic findings of infection. On the contrary, chronic bacterial prostatitis is characteristically a disease of the elderly. Systemic symptoms are typically not observed and the disease may mimic prostate cancer both clinically and radiologically [1].

On MRI, focal prostatitis tends to have low T2 signal with associated diffusion restriction. In addition, early phase arterial enhancement similar to prostate cancer may also be detected. For the aforementioned reasons, differentiation between focal prostatitis and prostate cancer may be highly challenging for both urologists and imaging specialists. Clinical findings may be helpful for accurate diagnosis as prostatitis shows fluctuating prostate-specific antigen (PSA) elevation, and decrease in PSA levels after antibiotic treatment. Radiologically, focal prostatitis tends to have less diffusion restriction on diffusion-weighted images. A lesion with well-defined, nodular morphology should raise suspicion of a tumor rather than focal prostatitis. Infectious-inflammatory lesions are generally linear and less mass-forming in shape rather than of rounded-nodular appearance, with most of them having ill-defined borders. Besides, prostatitis tends to have a lobar distribution or a diffuse involvement of the peripheral zone. Despite all efforts, targeted prostate biopsy is usually needed for definitive diagnosis [1–3].

Prostatic abscesses may be seen as a consequence of acute bacterial prostatitis. On MRI, abscesses demonstrate significant diffusion restriction similar to cancers. T2W images and DCE images may be helpful in these cases as abscesses typically have T2 hyperintense non-enhancing purulent content with thick, irregular, contrast-enhancing walls [1].

### Prostatic tuberculosis

Prostatic tuberculosis (TB) may be secondary to intravesical BCG instillation in patients with bladder cancer or due to systemic tuberculosis infection. The main differential diagnoses are prostate cancer and benign prostatic hyperplasia, as clinical signs and symptoms frequently overlap. PSA levels may be normal or high in patients with prostatic TB [4].

**Fig. 1:**
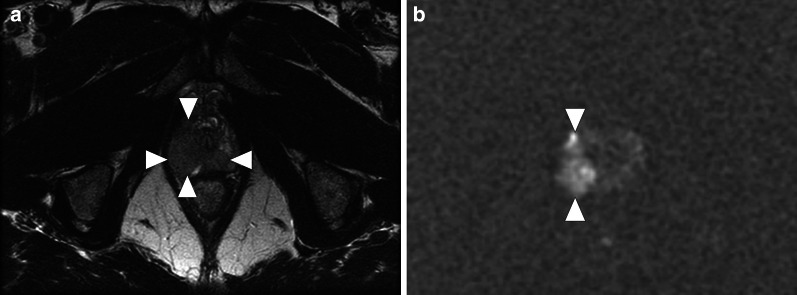
Granulomatous prostatitis after BCG therapy. A 62-year-old man with a history of bladder cancer treated with cystoscopic surgery and intravesical BCG therapy now presenting with deep pelvic pain and difficulty with urination. Axial T2-weighted image **a** of the prostate demonstrates a large hypointense lesion (arrowheads) on the right lateral and posterior peripheral zone of the prostatic apex. Axial diffusion-weighted image **b** (b = 2000s/mm^2^; 3 T) demonstrates pronounced diffusion restriction (arrowheads) concerning for prostate cancer. Fusion-guided transrectal biopsy confirmed tuberculous prostatitis with no evidence of malignancy

In a study of 6 patients with prostatic TB, two involvement patterns have been described: nodular and diffuse. In the nodular form, the TB nodules have been detected in both the peripheral and transition zones. The nodule diameters were reported to be between 4 and 18 mm and they are typically seen as hypointense on T2W images but isointense on T1W images. Diffusion restriction was also reported in these nodules (Fig. 1). All the nodules were found to be completely within the confines of the prostate gland with no extraprostatic extension. Imaging findings of the nodular form may closely mimic prostate cancer and differential diagnosis without histopathologic confirmation may be difficult or even impossible. Clinical and imaging findings may be inconclusive for the distinction of focal tuberculosis and prostate cancer, and targeted biopsy may be required to confirm the diagnosis [4].

Diffuse prostatic involvement is more common than the nodular form and both peripheral and transition zones have been reported to be involved. Diffusion restriction has been reported in these patients [4].

### Granulomatosis with polyangiitis

**Fig. 2:**
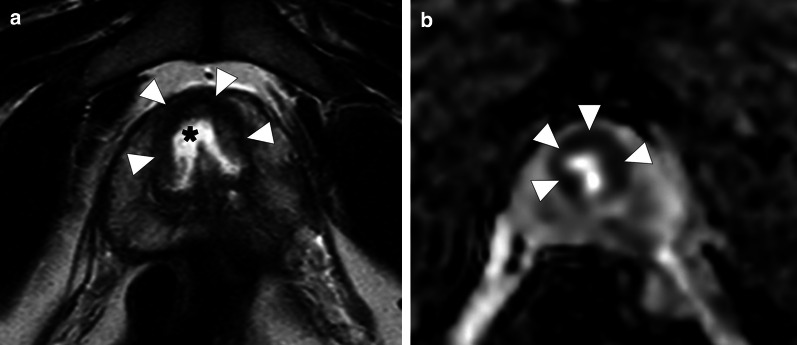
Prostatic involvement by granulomatosis with polyangiitis. A 31-year-old man with no known systemic disease presented with severe dysuria and hematuria. Serum PSA levels were also increased. Axial T2-weighted MR image **a** of the prostate demonstrates heterogeneous ill-defined, mostly hypointense lesion (arrowheads) in the prostate apex. A small cystic cavity appears in the central part of the lesion (asterisk). Axial ADC map (b = 800 s/mm^2^; 1.5 T) **b** shows diffusion restriction in the peripheral part of the lesion, suggestive of increased cellularity (arrowheads). Fusion guided transrectal biopsy revealed granulomatous prostatitis consistent with granulomatosis with polyangiitis

Prostatic involvement has been reported in 2.3–7.4% of cases of granulomatosis with polyangiitis [5, 6] with symptoms mimicking prostatitis and benign prostatic hypertrophy [5, 7, 8]. On MRI, foci of granulomatous prostatitis have low T2 signal intensity and low apparent diffusion coefficient (ADC), which makes it difficult to discriminate from prostate cancer [3]. Imaging diagnosis may be difficult as both prostate cancers and microbial prostatic abscesses can simulate vasculitic involvement of the prostate (Fig. 2).

### IgG4-related prostatitis

**Fig. 3:**
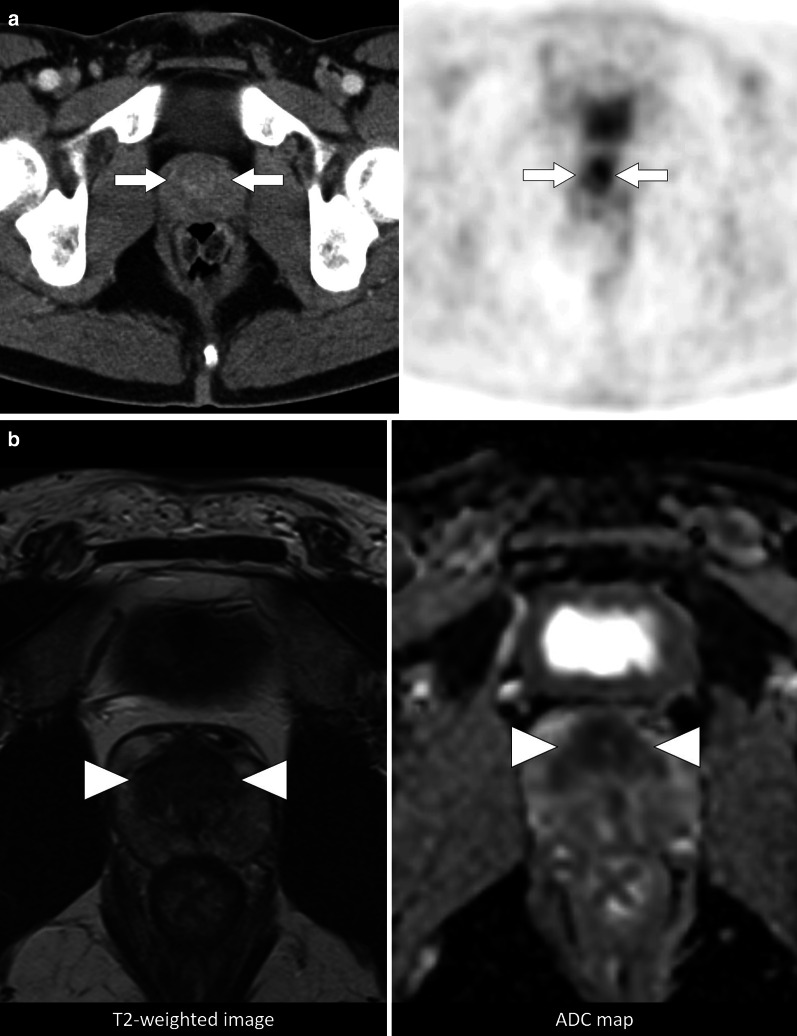
IgG4-related prostatitis. A 32-year-old man with well-established IgG4-RD underwent PET/CT for evaluation of disease activity. **a** Axial plane PET/CT images revealed an FDG-avid nodular lesion (arrows) in the prostate gland. Because of the patient’s medical history, prostatic involvement of IgG4-RD was considered primarily. Patient declined histopathological evaluation, and preferred close follow-up. **b** Follow-up multiparametric prostate MRI 4 years after the initial PET/CT examination showed no significant change in size in the 4-year interval. Axial T2-weighted image and ADC map (b = 1000 s/mm^2^; 1.5 T), respectively, demonstrate T2-hypointense lesion with low ADC values (arrowheads)

IgG4-related disease (IgG4-RD) is a fibroinflammatory disease characterized by multiorgan involvement and mass-forming lesions. The prostate involvement is very rare and called as IgG4-related prostatitis. Patients may present with enlarged prostate and obstructive urinary symptoms. Prostatic nodularity and asymmetry can be detected in rectal digital examination, and elevated PSA levels may be seen [9, 10]. Therefore, it can mimic malignancy from a clinical perspective. A few cases have been reported in the literature and its radiological findings have not been clearly defined yet. However, 18-F FDG PET/CT imaging may be helpful for differentiation from prostatic adenocarcinoma. While FDG-avid lesions are seen in IgG4-related prostatitis, FDG uptake is not expected finding for prostatic adenocarcinoma (Fig. 3) [11]. Although the history of IgG4-RD and the presence of other organ involvements such as autoimmune pancreatitis may be a clue, histopathological examination is required for definitive diagnosis.

## Testes

Primary testicular malignancies are among the most common neoplasms affecting the young male population. US has been shown to have a 92–98% sensitivity and a 95–99.8% specificity for testicular malignancy. On US, a solid testicular mass with internal vascularity is suggestive of a testicular tumor. Seminomas are usually homogeneous, hypoechoic masses and rarely demonstrate calcifications or cystic areas. Testicular non-seminomatous germ cell tumors are more heterogeneous in echotexture and generally appear more cystic as compared to seminomas [12, 13].

Several non-neoplastic conditions also may involve the testes, and familiarity with these processes is critical for correct diagnosis. MRI can be helpful to distinguish between benign and malignant testicular mass lesions, mainly recommended in cases of inconclusive US findings [14].

### Orchitis

Testicular infection, including orchitis, with or without abscess formation, may mimic a testicular neoplasm both clinically and radiologically. US examination may demonstrate hypoechogenicity in the testicular parenchyma due to diffuse edema in the acute phase of orchitis. This hypoechogenicity may become more localized as the inflammatory process evolve. Also, focal infection may show increased color flow at color Doppler imaging. For all these reasons focal orchitis may mimic a neoplastic process on US study. However, clinical findings of scrotal pain, fever, and elevated white blood cell count are more suggestive for orchitis over a testicular neoplasm [13].

The detection of associated hydrocele, pyocele, and scrotal edema are also among the other suggestive imaging findings for orchitis [36]. Follow-up US study may be performed 2–4 weeks after the initiation of antibiotic therapy to differentiate orchitis from a testicular tumor [12, 13].

### Mumps orchitis

Orchitis is the most common complication of mumps in pubertal and postpubertal males [15]. The virus directly invades the testicular tissue, causing edema and congestion of the seminiferous tubules with subsequent pressure necrosis and fibrohyalinosis. These parenchymal abnormalities cause the cessation of spermatogenesis. The seminiferous tubules become engorged with sloughing cells and debris [16]. Reported cases of infertility appear to be due to severe infections, with testicular atrophy developing months to a year after the infection [15].

**Fig. 4:**
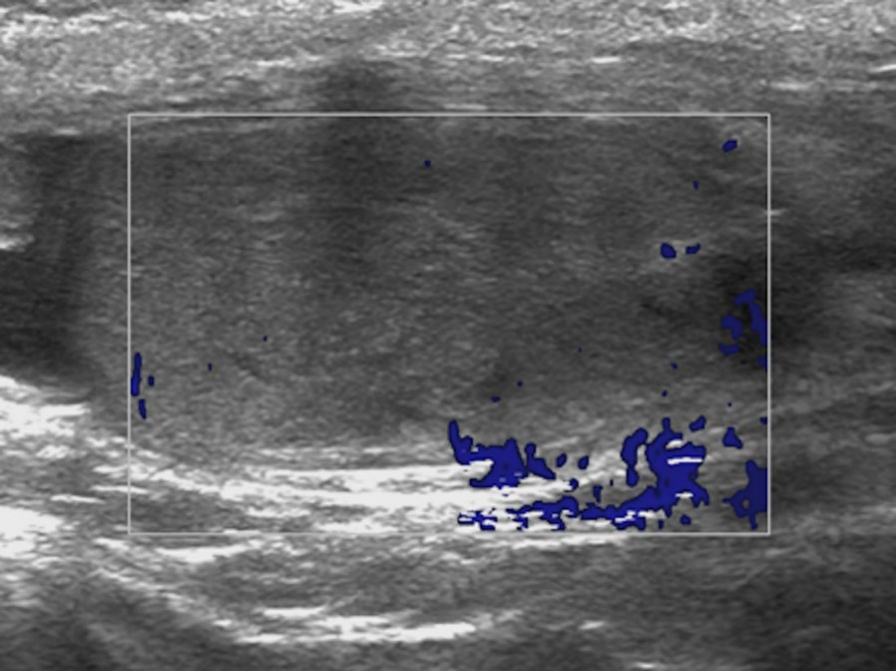
Mumps orchitis. A 32-year-old man referred from infertility clinic for general evaluation and mildly enlarged left testis. US exam demonstrated severely heterogeneous left testis with no evident vascularity within the testis parenchyma. The right testis was completely normal (not shown). Orchiectomy revealed severe and diffuse testicular fibrosis consistent with chronic mumps orchitis

US is the most commonly used imaging modality for evaluating patients with suspected mumps orchitis. Testicular enlargement and scrotal wall thickening are common findings in the early phase of infection [17]. Involvement of the epididymis is also common, around 85% [17]. Color flow Doppler may demonstrate increased vascularity in the testis in the early phases of the disease. Also, MRI may show early and homogenous contrast enhancement of the testicle [18]. On the chronic phase of the inflammation, the testis may undergo atrophy with the development of heterogeneous parenchymal echotexture (Fig. 4).

Severe parenchymal heterogeneity may mimic a diffusely infiltrative testicular tumor, so that differential diagnosis may be difficult without relevant clinical history and measurement of blood tumor markers.

### Tuberculous orchitis

Scrotal tuberculosis is seen in 7% of patients with TB, and tuberculous orchitis is even rarer. Underlying pathogenesis may be related to retrograde extension from the infected epididymis, prostate or seminal vesicles, or with hematogenous spread. Unlike bacterial orchitis, clinical findings of acute infection such as fever, leukocytosis, increased scrotal temperature, scrotal tenderness, and response to conventional antibiotic treatment are not expected in the initial presentation and the course of tuberculous orchitis [19]. From an imaging standpoint, the disease may mimic testicular tumors; however it should be in the differential diagnoses especially in cases with a history of tuberculosis. TB orchitis is rarely isolated and mostly seen in association with epididymo-orchitis. Therefore, the detection of coexisting epididymal involvement on imaging, characterized by heterogeneous hypoechoic epididymal enlargement, may be helpful in differentiating it from testis tumors [20].

As stated above, isolated testicular involvement appears to be extremely rare and is generally seen as a result of hematogenous spread [19].

**Fig. 5:**
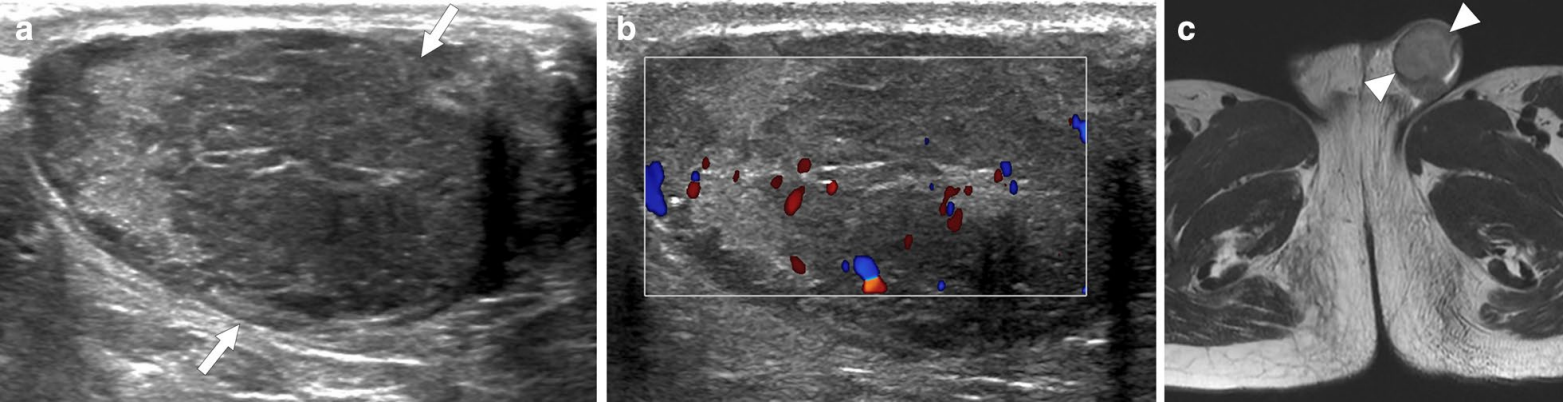
Tuberculous orchitis. A 13-year-old male with known inherited interleukin-12 receptor deficiency and recently diagnosed disseminated tuberculosis. Patient also complained of mild left testicular pain and asymmetric enlargement at his initial presentation. **a** Gray scale US image shows ill-defined heterogeneous hypoechoic areas (arrows) almost completely replacing the parenchyma. **b** Color flow Doppler US demonstrated mild-to-moderate vascularity within these hypoechoic areas. **c** Axial T2-weighted MR image shows enlarged left testis with irregular borders and low T2 signal intensity (arrowheads). Based on the clinical, microbiological and radiological findings, the putative diagnosis was tuberculous orchitis. Serial follow-up US examinations after the start of antituberculosis treatment demonstrated gradual testicular atrophy

On US, it may present as multiple small-sized hypoechoic nodules, or diffusely enlarged homogeneous or heterogeneous hypoechoic testicular parenchyma (Fig. 5). Scrotal skin thickening, complex hydrocele, scrotal abscesses, sinus tracts, or intrascrotal calcifications are other well-known radiological findings that may accompany tuberculous orchitis [20].

On MRI, generally, T2-hypointense lesions are seen in tuberculous orchitis, and it is thought that this finding may be secondary to chronic inflammation, fibrosis or calcification. Although the T1 signal is variable, high signal intensity has been described in most cases. Regarding the contrast enhancement of the lesions, a wide spectrum ranging from non-enhancement to strong enhancement have been reported [19]. Even in patients with suggestive radiological findings and clinical status, it may sometimes be difficult to differentiate TB orchitis from testicular tumors. Thorough microbiological evaluation and histopathologic examination are generally required to confirm the diagnosis.

### Xanthogranulomatous orchitis

Xanthogranulomatous orchitis (XGO) constitutes a very rare form of orchitis. It is characterized by extensive parenchymal destruction due to lipid-laden macrophage infiltration [21]. As the disease may present with painless scrotal mass, it may be clinically, and mostly radiologically, almost impossible to exclude testicular tumors. Epididymal obstruction, urinary infection, testicular ischemia are thought to be involved in the etiology of the disease [22]. An increased coexistence with diabetes has also been observed [23, 24]. In certain cases, urinary cultures may grow *E. coli* and *P. aeruginosa*, which may be suggestive of an infectious etiopathogenesis [22].

**Fig. 6:**
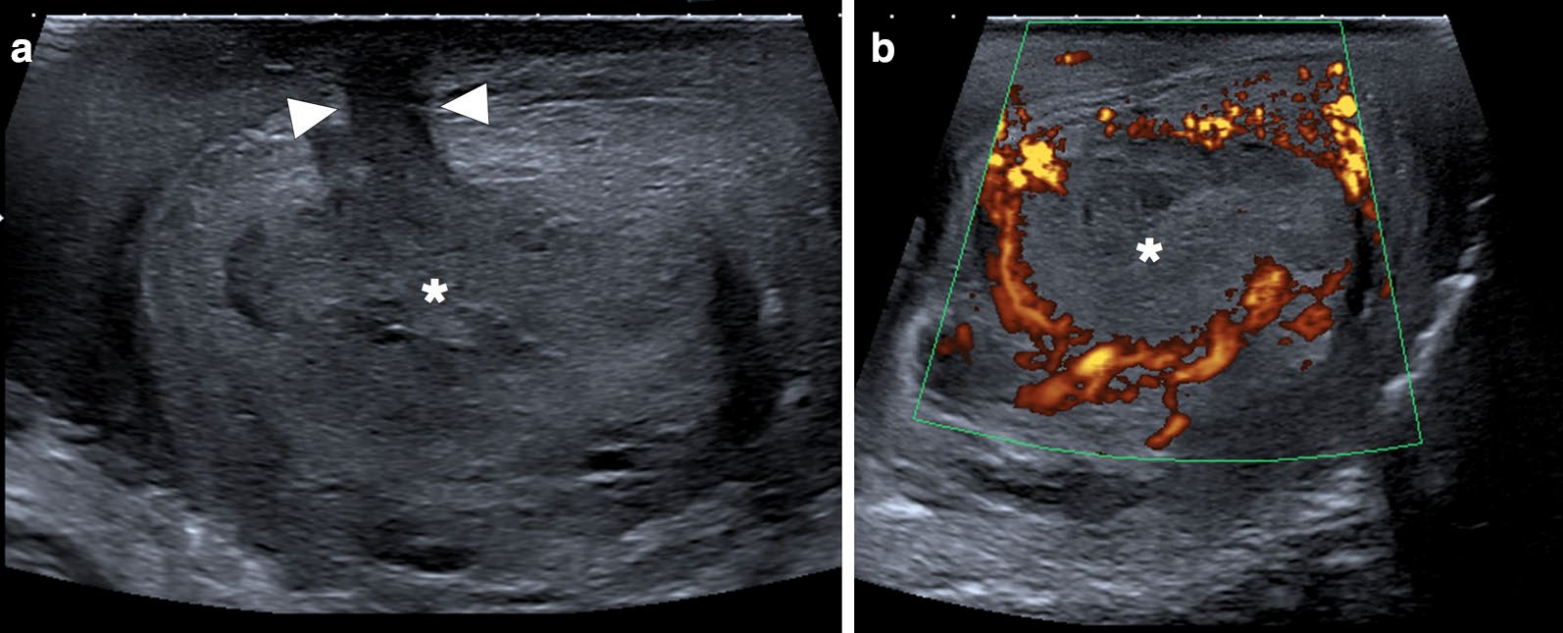
Xanthogranulomatous orchitis. A 19-year-old male presenting with left testicular mass and scrotal swelling. **a** Gray scale US image shows large ill-defined, heterogeneous mass within the testis (asterisk). Also noted was focal discontinuity in tunica albuginea (arrowheads). **b** Power Doppler US did not demonstrate any prominent vascularity within the lesion (asterisk) with marked peripherally increased blood flow. Radical orchiectomy was performed, and histopathological examination confirmed xanthogranulomatous orchitis. No causative microorganism was detected in tissue culture and other microbiological studies

In a limited number of cases, imaging findings have been described as large, heterogeneous masses with no apparent vascularity. Parenchymal calcifications, cystic changes, and epididymal involvement may also accompany testicular masses (Fig. 6) [22]. Imaging findings are mostly non-specific, and differential diagnoses include other infectious, inflammatory and neoplastic diseases of the testes. Moreover, XGO may coexist with testicular malignancies [25]. Radical orchiectomy can be performed for both definitive diagnosis and curative treatment [24].

### Testicular malacoplakia

Malacoplakia is another rare inflammatory disease of the testes which may sonographically mimic testis tumors. It is thought to develop secondary to chronic granulomatous inflammation due to chronic infection [21]. Malacoplakia is mostly detected in the bladder among the other organs in the genitourinary system [21]. However, testicular involvement has also been reported as anecdotal cases. Patients with testicular malakoplakia typically present with testicular enlargement or symptoms related to epididymoorchitis. On imaging, unilateral testicular enlargement, heterogeneous mass with cystic areas and intratesticular abscesses may be seen [14]. Histopathological examination is almost always required for differentiation from neoplastic lesions. In pathological examination, the presence of von Hansemann cells and Michaelis-Gutmann bodies (intracytoplasmic inclusion bodies) are pathognomonic [14].

### Testicular abscess

**Fig. 7:**
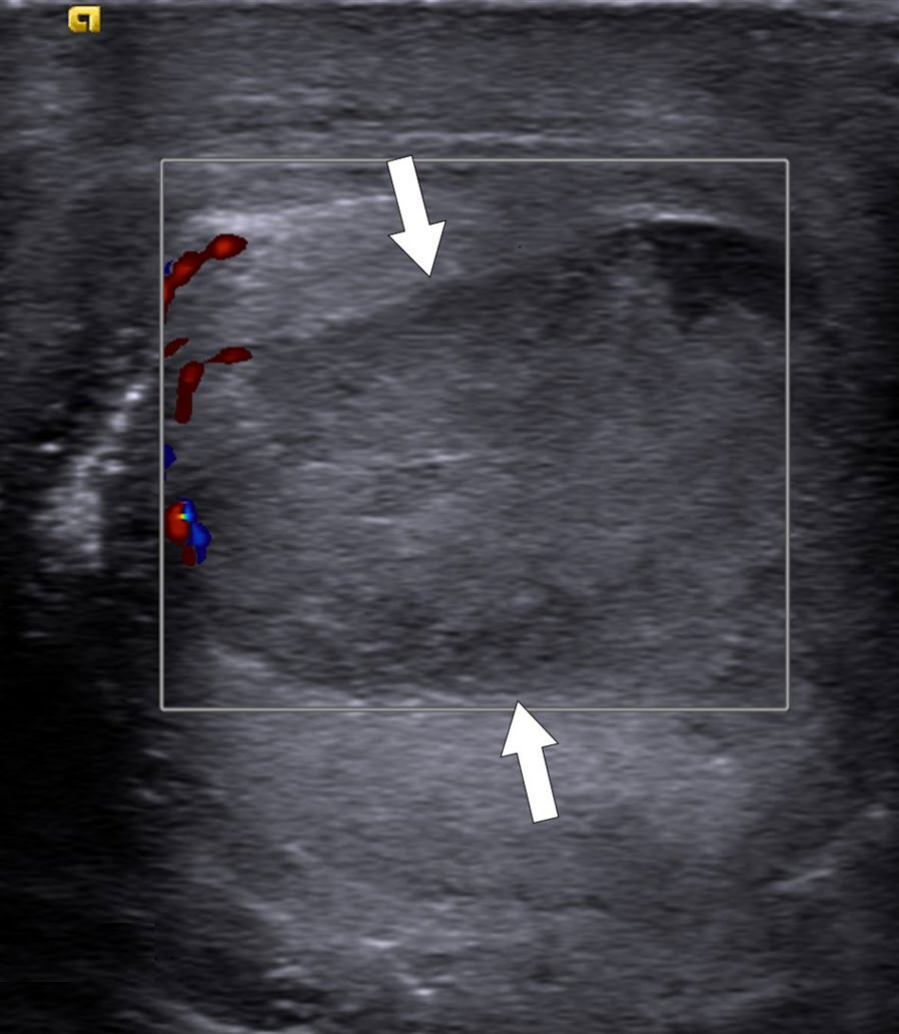
Testicular abscess. A 18-year-old male with known neurogenic bladder due to myelomeningocele presenting with high fever, right testicular swelling, and severe scrotal pain. Color Doppler US image shows heterogeneous, hypoechoic lesion (arrows) with no significant internal vascularity. Note was also made of scrotal skin thickening and edema. As the patient was almost septic, an emergent right orchiectomy was performed. Histopathological examination confirmed severe testicular inflammation and abscess formation

**Fig. 8:**
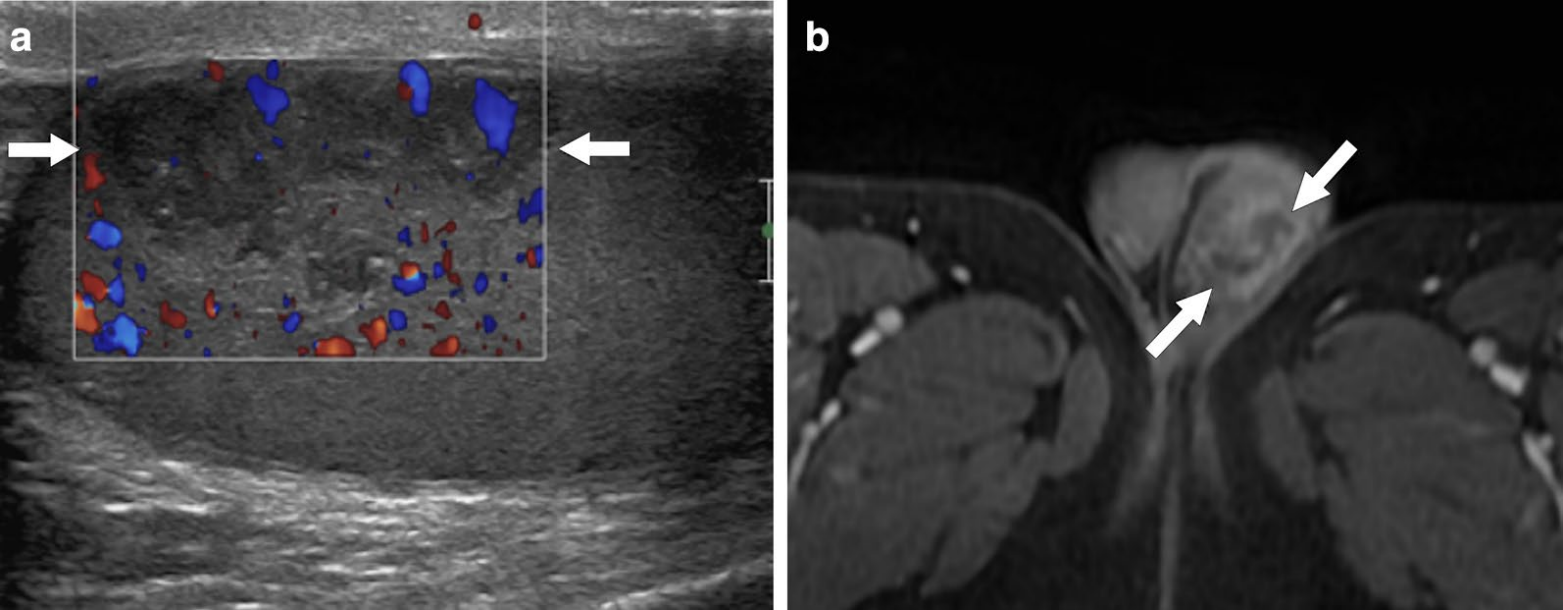
Testicular abscess. A 30-year-old healthy male with testicular pain and palpable scrotal mass. Clinical history and physical exam, apart from palpable left testicular mass, were unremarkable. **a** Color Doppler ultrasound image demonstrates a complex cystic mass with increased peripheral and mild central vascularity (arrows). **b** Postcontrast fat suppressed T1W axial plane image shows predominantly peripherally enhancing left testicular mass (arrows). Polymicrobial abscess was pathologically confirmed after orchiectomy. No obvious underlying risk factor could be discerned for testicular abscess

Testicular abscesses are usually a complication of bacterial epididymo-orchitis. In rare situations secondary infections of pre-existing hematomas or infarct areas have also been reported [26]. US is generally the first imaging modality used for detecting and diagnosing these abscesses. On US, testicular abscesses typically appear as hypoechoic lesions with ill-defined “shaggy” walls (Fig. 7) [27]. The content of the abscess is mostly heterogeneous and some movement within this content on gentle compression with the probe may be detected on real-time imaging. Perilesional increased testicular vascularity is frequently observed on color Doppler imaging, and the absence of Doppler signal within the abscess content is a helpful diagnostic sign favoring an abscess over a neoplastic process (Fig. 8).

**Fig. 9:**
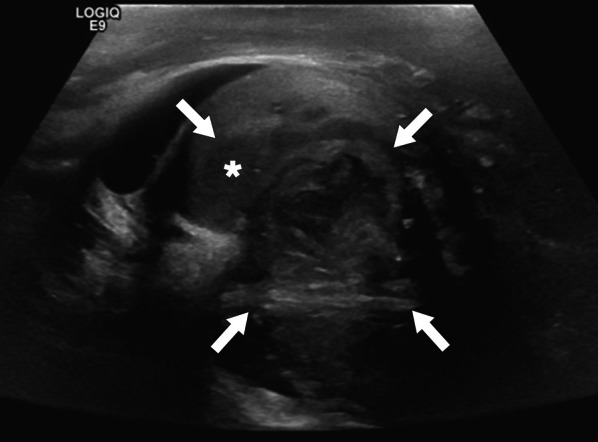
Mass-forming testicular abscess. A 69-year-old male who presented with recent onset of right testicular pain and palpable mass. Gray scale US image shows a heterogenous mass with ill-defined borders and central cystic areas. The imaging appearance was concerning for a paratesticular sarcoma (arrows) infiltrating testicular parenchyma (asterisk). Orchiectomy revealed no evidence of malignant disease but organized testicular abscess with paratesticular extension

**Fig. 10:**
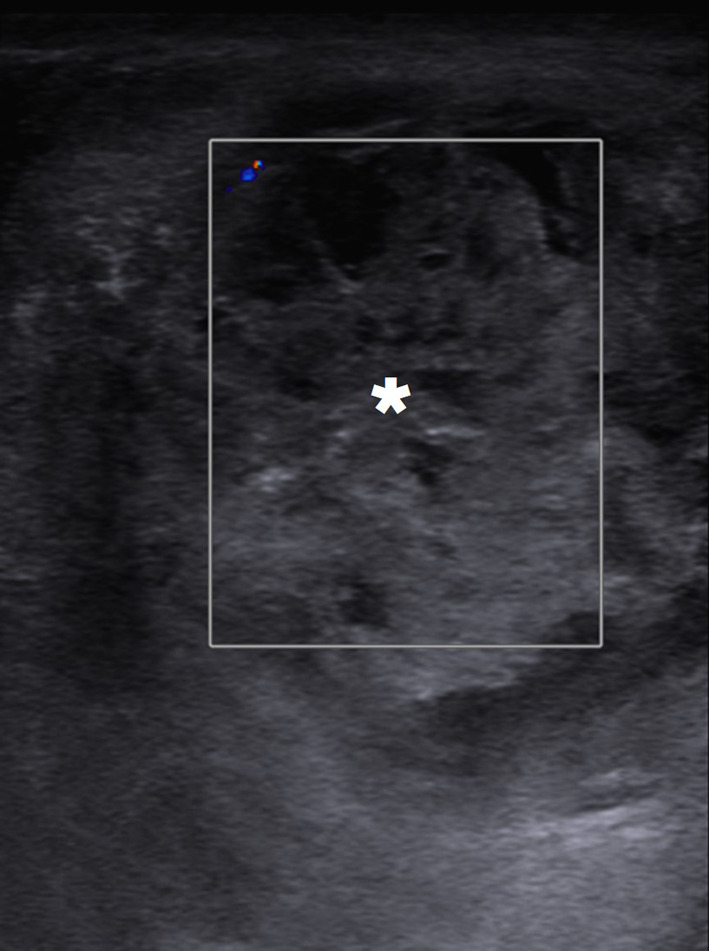
Testicular choriocarcinoma. A 25-year-old male with an unremarkable past medical history presenting with right testicular palpable mass and mild tenderness. Color Doppler US shows a large heterogeneous solid mass (asterisk) with no apparent internal vascularity on color Doppler study. Differential diagnoses based on US findings were necrotic testicular tumor, testicular hematoma or testicular abscess. As the concomitant serum beta-hCG levels were extremely high (150.000 mlU/mL), a testicular choriocarcinoma was considered as the leading diagnosis. Histopathological examination confirmed testicular choriocarcinoma

Abscesses with minimal cystic content may be difficult to differentiate from testicular neoplasms (Fig. 9). The differential diagnosis is further confounded by the propensity of some testicular cancers to undergo necrosis and cavitate, thus mimicking an abscess (Fig. 10). Relevant clinical history, presence of testicular pain, level of tumor markers and acute phase reactants may be helpful for correct diagnosis. On MRI, significant diffusion restriction and thick, irregular contrast-enhancing wall are typical features of abscesses regardless of the location. The absence of contrast enhancement within the lesion and the detection of perilesional hyperemia on contrast-enhanced MRI study may indicate an abscess [14]. In selected patients, percutaneous fine needle aspiration biopsy or rarely even surgery may be indicated.

### Testicular necrotizing vasculitis

**Fig. 11:**
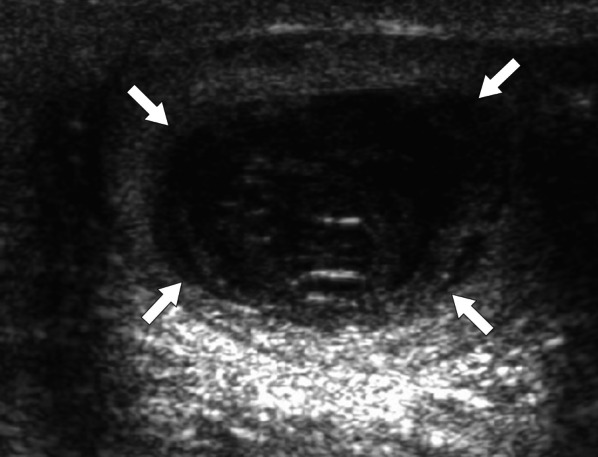
Testicular vasculitis. A 37-year-old male with known Churg-Strauss disease now presenting with new testicular mass. The patient was afebrile and did not report any pain. Ultrasound image demonstrates focal well-defined hypoechoic, centrally cystic mass, almost completely replacing the left testis (arrows). Orchiectomy revealed necrotizing vasculitis with no malignant cells

Testes may be affected in the course of several different types of vasculitis [28]. Polyarteritis nodosa is the most common. However, several other vasculitides including granulomatosis with polyangiitis, Churg-Strauss syndrome (Fig. 11), giant cell arteritis, and Henoch-Schonlein purpura may involve the testes [28]. In certain cases, the testes may be the only involved organ and image-based differential diagnosis may be challenging.

Testicular vasculitis is one of the great mimickers of neoplastic processes. The differential diagnosis may be even more difficult when parenchymal involvement is multifocal. In these patients, lymphoma or metastatic disease to the testes may be considered in the differential diagnosis. Subacute testicular torsion should also be considered [28].

**Fig. 12:**
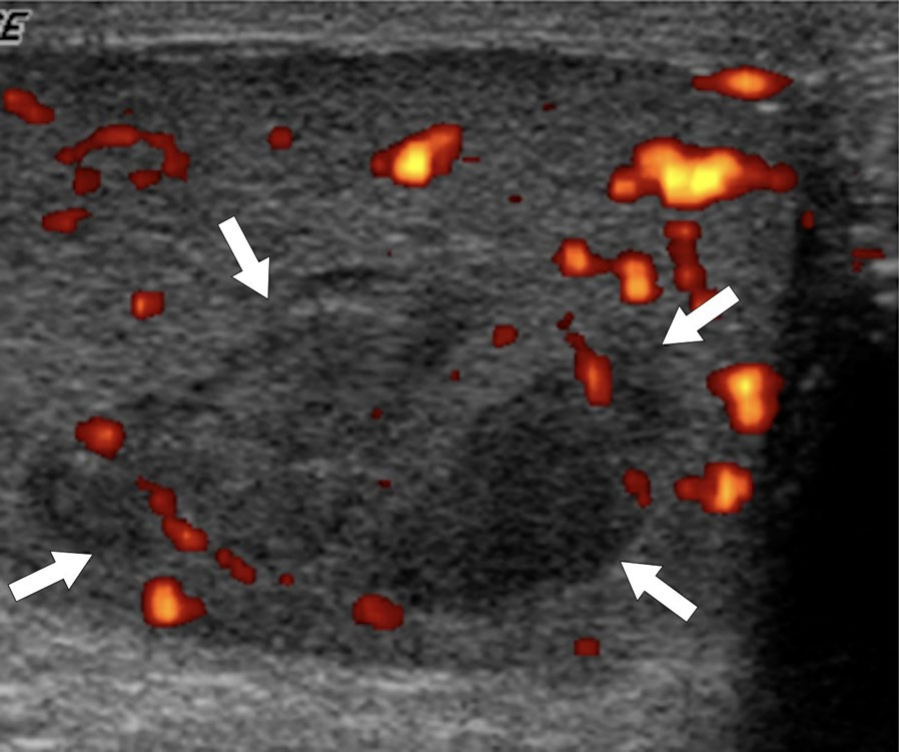
Isolated testicular vasculitis. A 35-year-old healthy male with progressive severe testicular pain and palpable mass on physical exam. Power Doppler ultrasound image demonstrates a hypoechoic peripherally located testicular mass (arrows) with mild increase in perilesional blood flow. Orchiectomy revealed necrotizing vasculitis and hemorrhagic infarct. There was no evidence of systemic vasculitis elsewhere in the body in the post-operative medical evaluation

US typically reveals a hypoechoic focal parenchymal lesion with heterogeneous internal echotexture. Color Doppler findings are variable (Fig. 12).

### Scrotal and testicular involvement in IgG4-RD

Testicular involvement is rare in patients with IgG4-RD. The involvement pattern may be subclassified as paratesticular and primary testicular involvement. Paratesticular fibrous pseudotumors are rare and may be either solitary or multiple. Histopathologically, they are composed of dense fibrous tissues with inflammatory infiltration of variable severity. Increased number of IgG4 positive plasma cells are typical for histologic diagnosis [29].

**Fig. 13:**
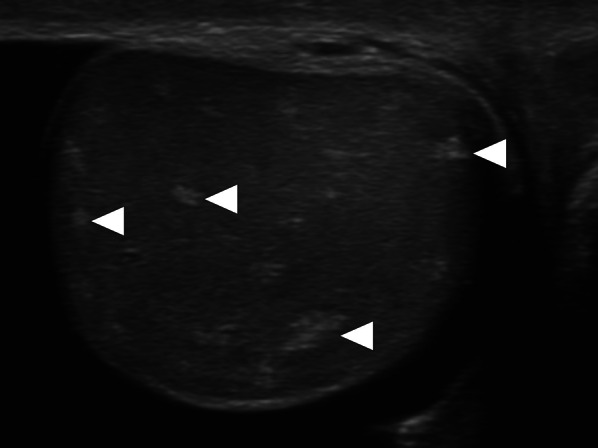
Testicular IgG4-related disease (IgG4-RD). A 35-year-old male with known systemic IgG4-RD presenting with new onset of blunt testicular pain and bilaterally enlarged testes. Ultrasound image demonstrates multiple irregular shaped hyperechoic lesions (arrowheads) scattered within both testes. As the process was bilateral and the patient had known IgG4-RD, a non-operative approach was elected. Intense medical treatment provided immediate relief and follow-up US study 6 months after the first US revealed stable findings

Testes themselves may also become involved in the course of IgG4-RD (Fig. 13). There is not much information in the imaging literature regarding imaging patterns of testicular involvement due to the rarity of the disease. In one case report, the disease manifested with a hypoechoic focal mass within the testicular parenchyma and final orchiectomy confirmed IgG4-RD rather than a testicular tumor [30].

### Testicular sarcoidosis

Sarcoidosis is a multisystemic disease characterized by noncaseating granulomas. It is most commonly seen among African Americans and the people of northern Europe. Involvement of male genitourinary system in sarcoidosis is uncommon but may be seen in 0.2% of patients. Epididymides and the testes are most commonly involved, followed by spermatic cord and prostate. Epididymal involvement is bilateral in one-third of the cases and this involvement is commonly asymptomatic. However, pain or the detection of a scrotal mass may be seen in a certain subset of the patients. Epididymal involvement may appear as a solid lesion in the epididymis, and surgery may be needed to rule out malignancy [31]. Isolated involvement of the testes is very rare and may mimic a testicular tumor.

Sarcoidosis should be considered in patients who have multiple lesions involving the testis and epididymis. Sonographic findings may mimic a primary testis tumor or lymphomatous involvement, and the differential diagnosis may be extremely difficult especially in patients who do not have a history of previously diagnosed sarcoidosis. Elevated serum ACE levels may also act as a supportive finding for correct diagnosis [27, 32, 33].

**Fig. 14:**
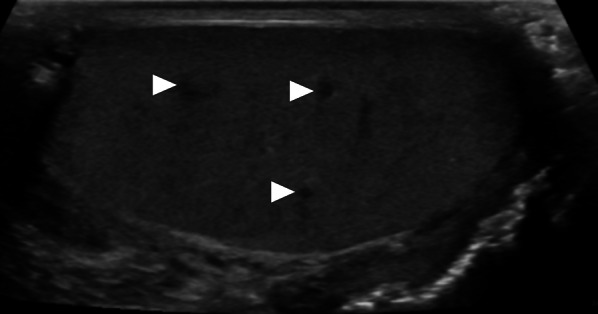
Testicular sarcoidosis. A 46-year-old male with known sarcoidosis presenting with mild testicular pain. Longitudinal plane gray-scale US image shows several subcentimeter hypoechoic nodules scattered throughout the testis parenchyma (arrowheads). These lesions were presumed to represent testicular involvement of the disease and were found to be stable on follow-up US studies (not shown)

Sonographically the typical imaging clue is the detection of multiple hypoechoic lesions (with or without involvement of the epididymides) scattered throughout the testes (Fig. 14). However, presentation with solitary mass may also be seen [33]. Color Doppler imaging usually does not demonstrate apparent vascularity within these lesions and that may be helpful for differentiating from primary tumors of the testis [34].

### Testicular hematoma

Intratesticular hematomas can be seen in trauma cases and the diagnosis is generally straightforward in these patients. However, incidental diagnosis of testicular tumors after trauma is not rare and has been reported in 10–15% of patients with a history of scrotal trauma [35]. Follow-up imaging is necessary in these patients to document resolution, exclude incidental tumors and detect secondary complications related to intratesticular hematomas [35, 36]. Surgical exploration may be needed in large-sized hematomas to prevent testicular necrosis and compressive parenchymal atrophy [35].

Testicular hematomas can be seen as single or multiple lesions of variable sizes. Sonographic findings tend to vary depending on the age of the hematoma. Characteristic finding is the detection of an iso-hyperechoic mass in the acute phase and differentiating the hematoma from the healthy parenchyma may be difficult. Unlike neoplastic masses, internal vascularity is not expected in hematomas with color Doppler US [36]. However, peripherally increased vascularity can be seen in infected hematomas [35]. In the resolution phase, hematomas may appear as hypoechoic lesions with gradual decrease in size over serial follow-up examinations. Concomitant scrotal trauma findings such as tunica albuginea disruption, testicular fracture, hematocele and scrotal wall hematoma can facilitate the differential diagnosis [35].

**Fig. 15:**
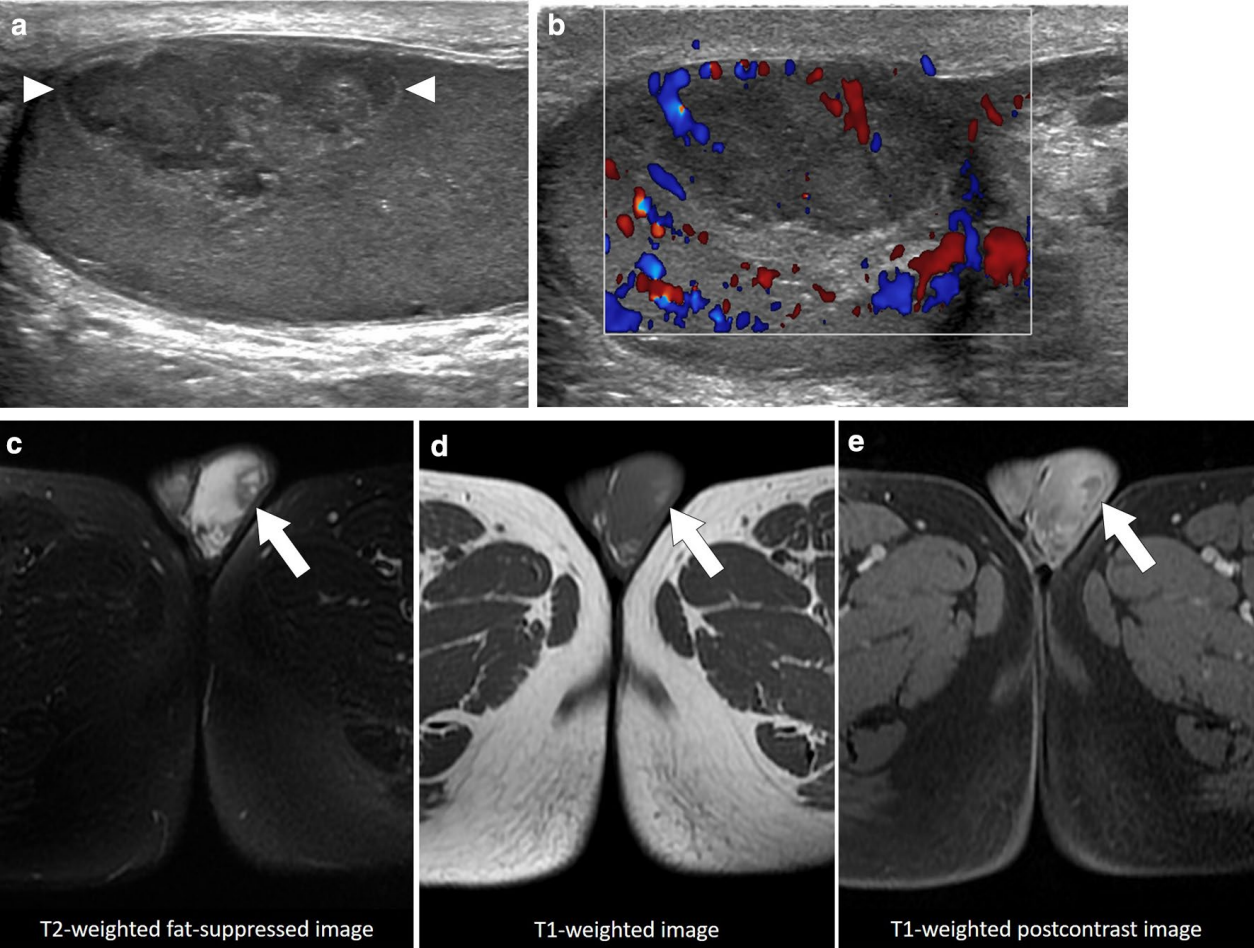
Testicular hematoma. A 30-year-old male presenting after a fall from height for which he did not seek any medical care. Ten days after the initial event, he presented with a palpable left testicular mass. **a** Gray scale US shows a heterogeneous hypoechoic lesion in the left testis (arrowheads). **b** There was mild internal vascularity within this lesion on color Doppler US exam. Hematoma was considered as the initial diagnosis but as testicular tumor could not be ruled out an MRI order was placed. **c-d** Axial plane T2W fat-suppressed and T1W images show high T1 and T2 signal intensities within the central part of the lesion (arrows) which was thought to represent hemorrhage in subacute phase. The peripheral low T2 signal intensity was considered hemosiderin deposition. **e** Axial plane postcontrast MR image shows no internal enhancement within the central part (arrow). During the follow-up, the lesion completely regressed, leaving mild testicular atrophy (not shown)

Temporal variation of the hematoma morphology may be better detected with MRI. While T1 hyperintensity is expected in the subacute period, a low T2 signal due to hemosiderin deposition is expected in the chronic phase. Contrast-enhancement within the hematoma is not expected on postcontrast T1W images (Fig. 15) [37, 38].

### Segmental testicular infarction

Segmental testicular infarction (STI) is a rare condition. Testicular pain is the most common mode of clinical presentation. It is mostly idiopathic, however, several other disorders such as acute epididymo-orchitis, trauma, vasculitis, and hematologic disorders may be the underlying reason. It is most commonly seen in patients who are between 20 and 40 years of age [27].

**Fig. 16:**
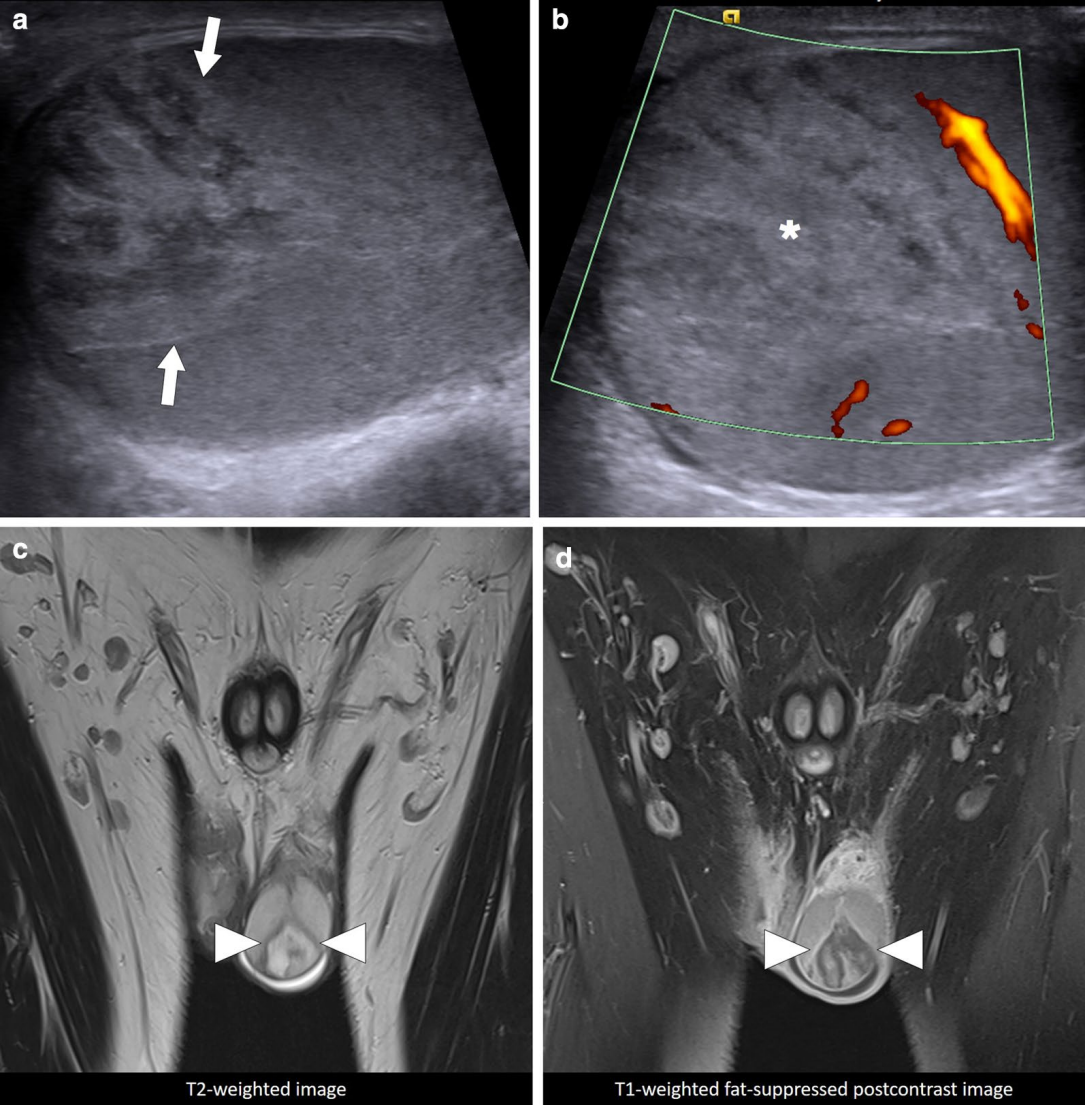
Segmental testicular infarction. A 32-year-old male with known Behcet’s disease presenting to the ER with left testicular pain. There was no history of trauma. **a** Gray scale US shows a triangular heterogeneous hyperechoic lesion (arrows) within the left testis. **b** On Power mode Doppler US, there was no vascularity within this focal area (asterisk). **c** Coronal plane T2W MR image shows triangular heterogeneous hyperintense lesion with a T2 hypointense rim. **d** Coronal plane postcontrast MR image demonstrates a hypoenhancing, wedge-shaped parenchymal area with sharp borders. Imaging findings were considered to be consistent with segmental testicular infarction, likely related to Behcet’s disease. Patient was placed on supportive treatment, to which he responded well. Serial follow-up US examinations revealed almost-complete resolution within 3 weeks after the initial event

On gray-scale US the infarcted area appears more heterogeneously hypoechoic as compared to the background parenchyma. Color Doppler US typically shows absence of vascularity in the infarcted segments. In the early phase they generally appear as a solid lesion; however, the absence of vascularity may favor the diagnosis of STI over a tumor. The infarcted segment is typically wedge-shaped with the vertex pointing towards the testicular mediastinum (Fig. 16). Follow-up imaging after supportive treatment is mandatory for ruling out a malignancy. Predisposing factors should also be sought in relevant clinical context [39].

MRI may be used in patients where differential diagnosis may not be made with US. On T2W images, STIs are typically seen as heterogeneous parenchymal areas with relatively well-defined borders. Hemorrhagic elements may be detected as hyperintense signal areas within the infarcted area on precontrast T1W images. The absence of enhancement within the infarcted area is typical on postcontrast T1W images. Rim enhancement may be detected in the periphery of the infarcted area in the majority of patients [27, 39].

### Leydig’s cell hyperplasia

Leydig's cell hyperplasia (LCH) is a rare, benign condition. It is histologically characterized by small, multifocal, and frequently bilateral parenchymal nodules in both testes. On microscopic examination, the hyperplastic Leydig cells are seen infiltrating between the seminiferous tubules. The size of these non-neoplastic nodules ranges from 1 to 6 mm in size [27, 40]. These patients are mostly asymptomatic and serum tumor markers are characteristically normal. However, lymphoma, leukemia, metastatic diseases, and multifocal primary testicular neoplasms may be considered in differential diagnosis in relevant clinical settings. An association between LCH and Klinefelter syndrome has been reported [41]. The patient’s clinical history and bilaterality may be helpful to differentiate LCH from a testicular neoplasm [27, 40].

**Fig. 17:**
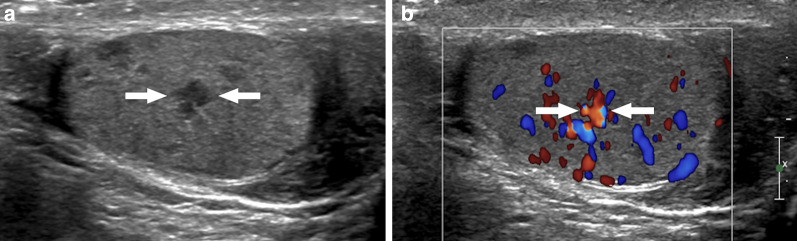
Leydig’s cell hyperplasia. A 34-year-old male presenting with infertility underwent testicular US exam for primary evaluation. **a** Gray-scale US image shows several hypoechoic lesions scattered throughout the parenchyma in both testes. The largest lesion was in the right testis (arrows). Both testes were deemed to be small in size when patient’s age was considered. **b** Color Doppler US image demonstrates intense vascularity within the largest lesion. Provisional diagnosis of Leydig’s cell hyperplasia was made. Genetic analysis confirmed Klinefelter’s syndrome

On US, the imaging findings are variable and the parenchymal nodules may appear as hypo- or hyperechoic. Vascularization may be detected within these nodules on Doppler US examination [40] (Fig. 17).

MRI may detect more nodules as compared to US and characterization may be more successful with this modality. On T2W images the nodules are typically hypointense with avid contrast enhancement on postcontrast T1W images [40].

### Testicular adrenal rests

Testicular adrenal rest (TAR) is a rare benign non-neoplastic testicular lesion. They originate from primordial adrenal gland cells that remained within the developing gonad during the fetal life [14]. These primordial cells may enlarge under high serum levels of adrenocorticotropic hormone (ACTH) and mimic neoplastic parenchymal masses. This clinical situation is mostly apparent in patients with congenital adrenal hyperplasia (CAH) and Cushing's syndrome [42]. TAR is relatively common in patients with CAH and has been detected in approximately 40% of these patients [42].

**Fig. 18:**
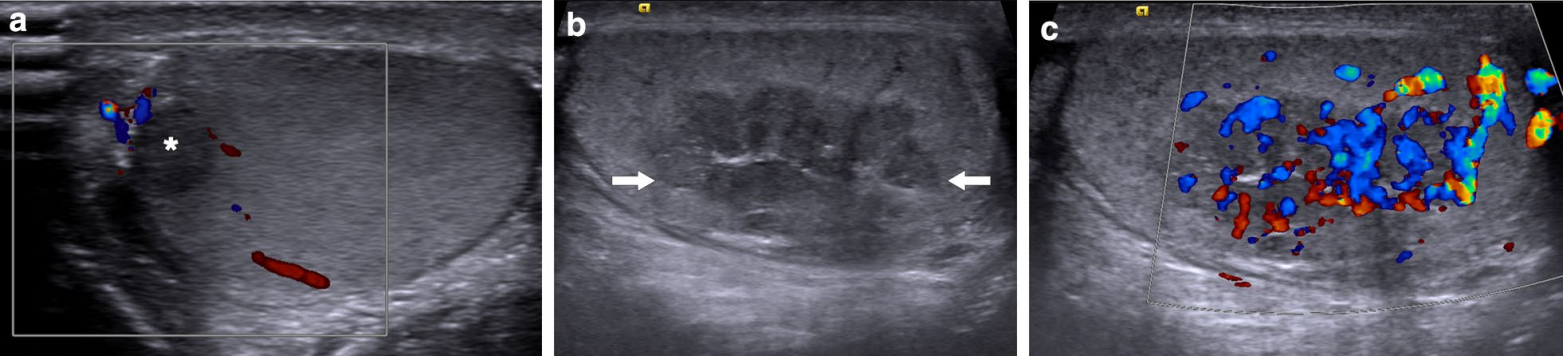
Two different patients with known history of congenital adrenal hyperplasia. **a** Gray scale and color Doppler US images show round lesions in both testes in 13-year old male. The most prominent lesion appears as a focal hypoechoic lesion within the mediastinum of the right testis (asterisk). There was no significant internal vascularity on color Doppler US. Based on the patient’s history and imaging findings, testicular adrenal rests was considered. Serial US exams over 2-years confirmed stability of testicular lesions. **b-c** Gray scale US **b** shows bilateral lobulated, large-sized hypoechoic lesions within the mediastinum testis in 15 years-old male. The largest lesion was detected within the right testis as a predominantly hypoechoic, heterogenous lesion (arrows). **c** On color Doppler US, there was intense internal vascularity within the lesion. These lesions were also considered to be consistent with testicular adrenal rests

On US, they are typically seen in both testes as multiple, hypoechoic nodular lesions with sharp rounded margins in the mediastinum testis (Fig. 18). They tend to appear as heterogeneous hyperechoic masses as they get larger in size. Posterior acoustic shadowing may also be observed in lesions with abundant fibrosis [43]. On color Doppler US, normal testicular vessels can pierce through these lesions without any distortion, which may serve as a clue to non-neoplastic nature of these lesions [43]. On MRI, they are generally seen as iso-hyperintense on T1W, hypointense on T2W, with avid enhancement on postcontrast T1W images [43].

As these lesions may negatively affect spermatogenesis by reducing gonadotropin levels or obstructing the seminiferous tubules, they may over the long term cause infertility in some patients [43]. Glucocorticoid therapy, rather than surgery, has been recommended for the management of TARs [14]. After glucocorticoid therapy regression of the lesions may be seen, which supports the diagnosis [14].

### Tubular ectasia of rete testis

Tubular ectasia of rete testis (TERT) is a benign condition caused by the total or incomplete occlusion of the efferent ducts [44]. Patients present with testicular pain and palpable scrotal mass on clinical examination. It is rare in young patients and most are over the age of 45 [45]. Bilateral involvement is not rare and may be seen in approximately 30–70% of patients [45, 46]. TERT has been reported in association with previous scrotal surgeries, vasectomy procedure and presence of spermatocele [45].

**Fig. 19:**
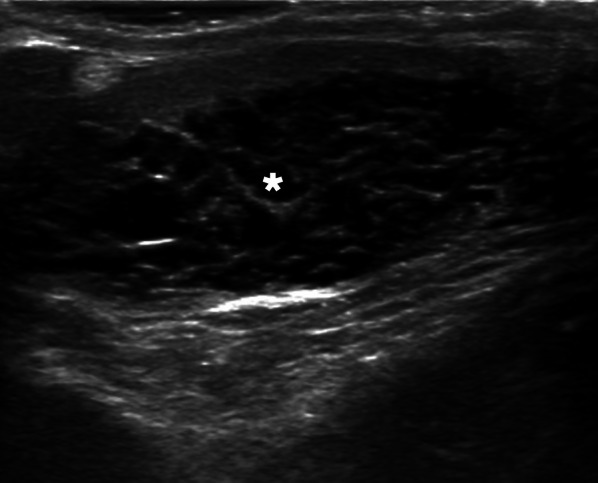
Tubular ectasia of rete testis. A 57-year-old male with a history of bilateral vasectomy now presenting with a right testicular mass. Physical exam revealed bilateral small testes with a questionable palpable mass on the right side. Gray scale US image demonstrates multiple cystic-tubular anechoic structures completely replacing the mediastinum testis (asterisk) in the right. There were also similar findings in the left testis, to a lesser extent (not shown). Based on patient’s history the findings are consistent with extensive rete testis ectasia in the right testis. Follow-up imaging studies demonstrated stability of the findings over 2 years (not shown)

On US, TERT is seen as multiple cystic-tubular anechoic structures replacing the mediastinum testis with no obvious mass effect (Fig. 19). Vascularity is not expected in color Doppler US. On MRI, TERT is observed as a T2 hyperintense cystic structure with no enhancement [45].

Although its location and imaging findings are typical, it is important to differentiate from semisolid testis malignancies containing cystic degeneration areas that may involve rete testis, such as cystadenocarcinoma of the rete testis [46]. Caution should be exercised when diagnosing TERT as post-obstructive dilation of the seminiferous tubules may be observed in testicular tumors [45].

Calcification is not an expected finding in TERT. Additionally, the detection of solid component, heterogeneous internal structure and internal vascularity on Doppler US should be alarming for a malignant process over TERT [45].

### Cystic dysplasia of rete testis

**Fig. 20:**
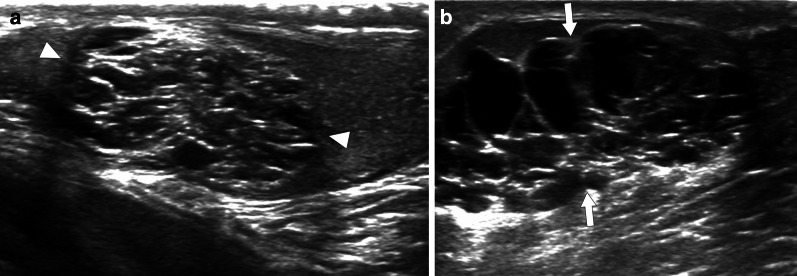
Cystic dysplasia of the rete testis in two different patients with known ipsilateral multicystic dysplastic kidney. **a** A 3-year-old boy with palpable right testicular mass. Gray scale testis US showed well-defined multicystic lesions within the right mediastinum testis. The lesion contains serpiginous tubular anechoic areas with intervening echogenic septations (arrowheads). No internal vascularity was detected on color flow Doppler US exam (not shown). Cystic dysplasia of rete testis was considered due to patient demographics and history. The lesion was stable on his last follow-up US exam 2 years after initial presentation. **b** A 7-year-old boy presenting with left testicular swelling. Gray scale US image shows a large multicystic lesion almost completely replacing the left testis parenchyma (arrows). Again the patient demographics, history and imaging findings were consistent with cystic dysplasia of the rete testis. Findings were stable on serial follow-up US exams

Cystic dysplasia of the rete testis is a rare and benign congenital entity observed in the pediatric age group. It may present with unilateral painless scrotal swelling and mimic testicular malignancies [47]. It is frequently observed together with ipsilateral renal anomalies such as renal agenesis, multicystic dysplastic kidney, vesicoureteral reflux or ureteral duplication [48]. On ultrasound, a unilateral multicystic lesion, consisting of cysts of varying sizes and partially compressing the surrounding parenchyma is seen in the mediastinum testis (Fig. 20). In some cases, testicular atrophy may develop secondary to the parenchymal compression [48].

Lesion location, patient age, sonographic findings, negative tumor markers, and accompanying ipsilateral genitourinary anomalies are important clues for diagnosis. Although the definitive treatment is surgical excision with testis-sparing surgery, “watch and wait” approach is generally recommended [48].

### Paratesticular region

The paratesticular region includes the spermatic cord, epididymis, tunica vaginalis and embryological remnants [49]. While the majority of intratesticular lesions are malignant, a vast majority of paratesticular lesions are benign [27]. Therefore, accurate determination of the lesion location is of great importance for correct patient management.

**Fig. 21:**
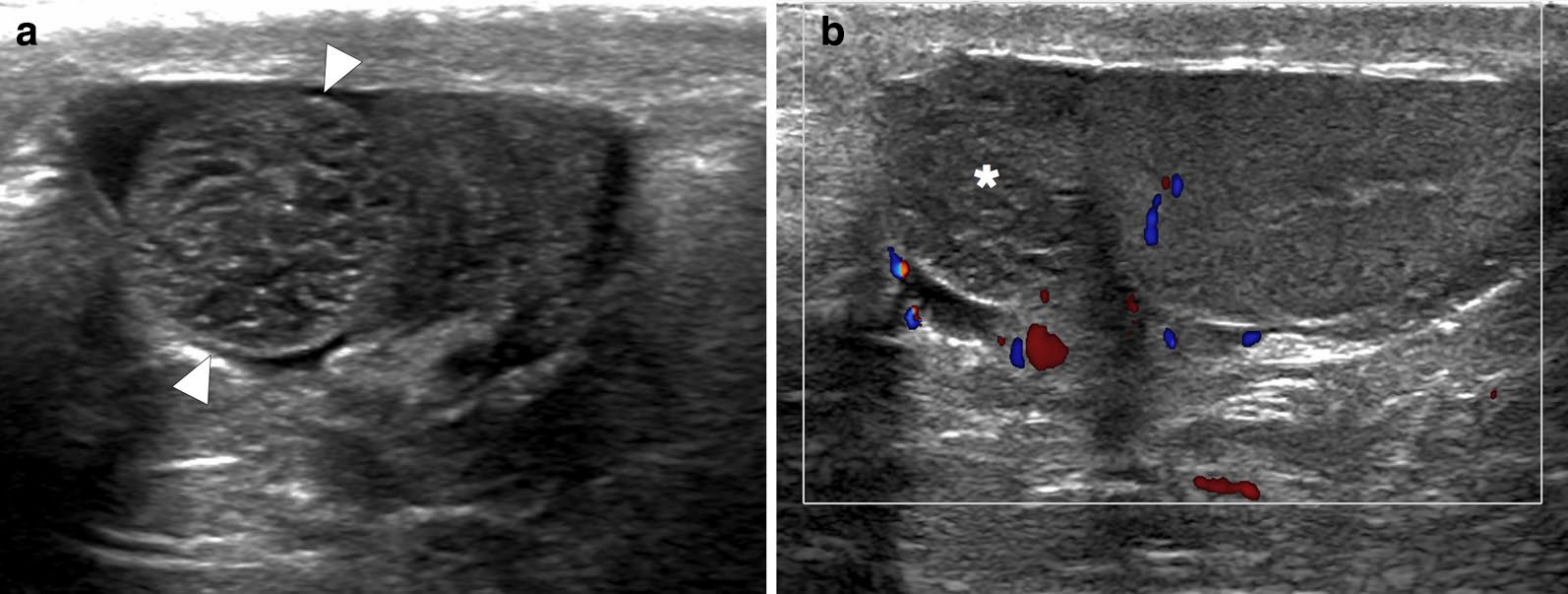
Torsion of the appendix testis. A 9-year-old boy who presented to ER with left-sided scrotal pain which started 3 days before presentation. Physical examination revealed left testicular tenderness and a small, firm scrotal mass. **a** Gray scale US image shows a round mildly heterogeneous solid-appearing extratesticular lesion adjacent to the upper pole of left testis (arrowheads). Minimal hydrocele and scrotal wall thickening were also noticed on US. **b** Color flow Doppler US shows the absence of vascularity within the lesion (asterisk). As clinical history was more suggestive for torsion of the appendix testis, the patient was placed on supportive treatment. He responded well to treatment, and his symptoms completely resolved within a week

On US, it is important to be aware of tumor mimickers in this area to prevent any unnecessary surgical or medical intervention. Conditions such as scrotal hernia, hematocele, epididymitis, and testicular appendage torsion can frequently present with a paratesticular mass. However the differential diagnosis of these conditions with their clinical and radiological features is generally straightforward (Fig. 21). On the other hand, some rare inflammatory diseases and congenital anomalies involving paratesticular region may cause diagnostic confusion.

### Paratesticular inflammatory pseudotumor

Inflammatory pseudotumor (IPT) is a general term that comprises benign and possibly reactive soft tissue lesions of unknown cause. In the literature, IPT is known by many different names such as fibrous pseudotumor, inflammatory myofibroblastic tumor, plasma cell granuloma, and pseudosarcoma. It is more common in young adults with a male preponderance. It can affect many different organ systems such as the lungs, extremities, gastrointestinal and genitourinary systems [50].

Paratesticular IPTs are uncommon lesions and they constitute 6% of all paratesticular lesions [51]. Most of them originate from tunica vaginalis; however, tunica albuginea, spermatic cord and epididymal involvement may also be seen. They are characterized by chronic fibroinflammatory soft tissue lesions thought to be secondary to previous surgery, trauma, infection or inflammation [50]. The presence of IgG4-expressing plasma cell has also been reported in some IPTs, which is thought to be suggestive for localized form of IgG4-RD [52].

**Fig. 22:**
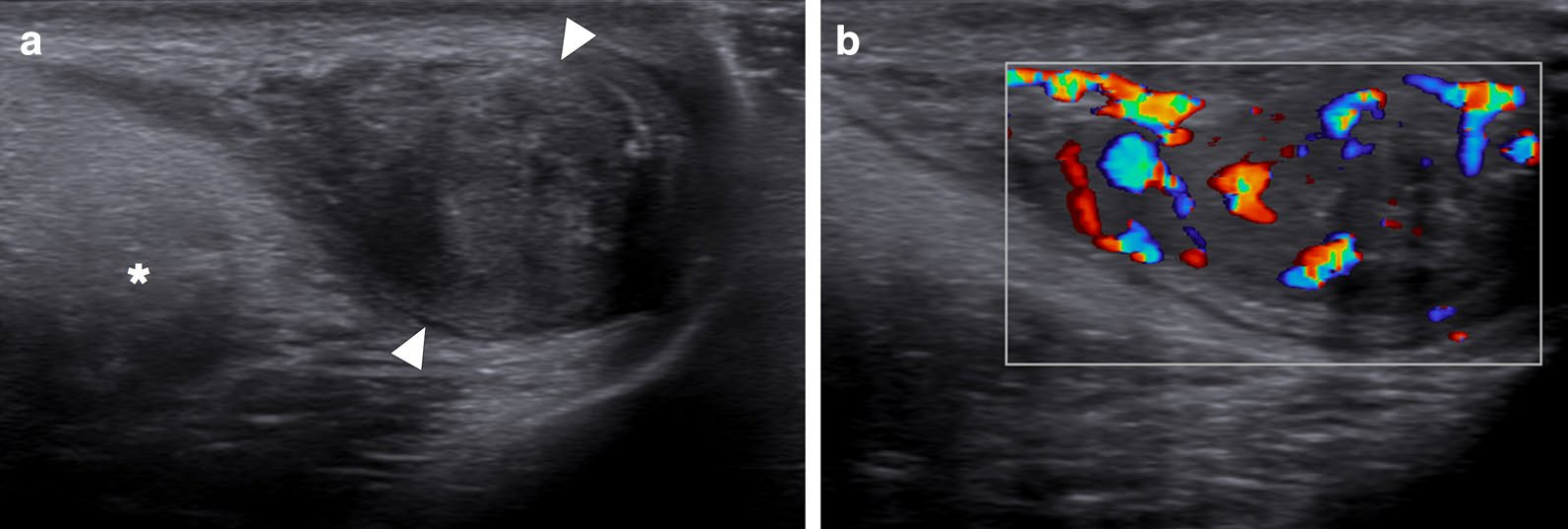
Paratesticular inflammatory pseudotumor. A 11-year-old boy who presented to ER with painless left scrotal swelling. **a** Gray scale US image demonstrates a heterogeneous, predominantly hypoechoic extratesticular lesion (arrowheads), adjacent to the lower pole of the left testis (asterisk). Also noted were a few scattered intralesional calcifications. **b** Color Doppler US exam shows widespread internal vascularity. The findings suggest an intrascrotal-extratesticular tumor, such as rhabdomyosarcoma. Surgical removal of the tumor, with preservation of the left testis, instead found paratesticular inflammatory pseudotumor

Patients usually present with a painless scrotal mass and their pre-operative diagnosis can be quite difficult. IPTs may mimic neoplastic lesions both clinically and radiologically. Their echogenicity on ultrasound may vary depending on the cellular component and the amount of fibrosis [50]. Posterior acoustic shadowing and intralesional calcification may be seen on US. Mild-to-moderate internal vascularity is expected on Doppler US (Fig. 22) [53]. On MRI, low signal on both T1W and T2W images and gradual contrast enhancement can be seen due to the fibrotic component [27]. Surgical resections are considered curative because of the benign nature, and testis-sparing surgery is recommended whenever possible [27, 50].

### Paratesticular tuberculoma

Genitourinary tuberculosis commonly involves epididymis. However, isolated epididymal involvement is a rare and difficult-to-diagnose entity, which is generally observed in the young adult population between the second and fourth decades of life [54]. It is mostly associated with immunosuppressive conditions such as HIV infection, or intravesical BCG instillation for superficial bladder cancer [54]. Although the heterogeneous hypoechoic enlarged epididymis parenchyma is the well-known US finding, epididymal tuberculosis may rarely present with paratesticular tuberculomas [20, 55].

**Fig. 23:**
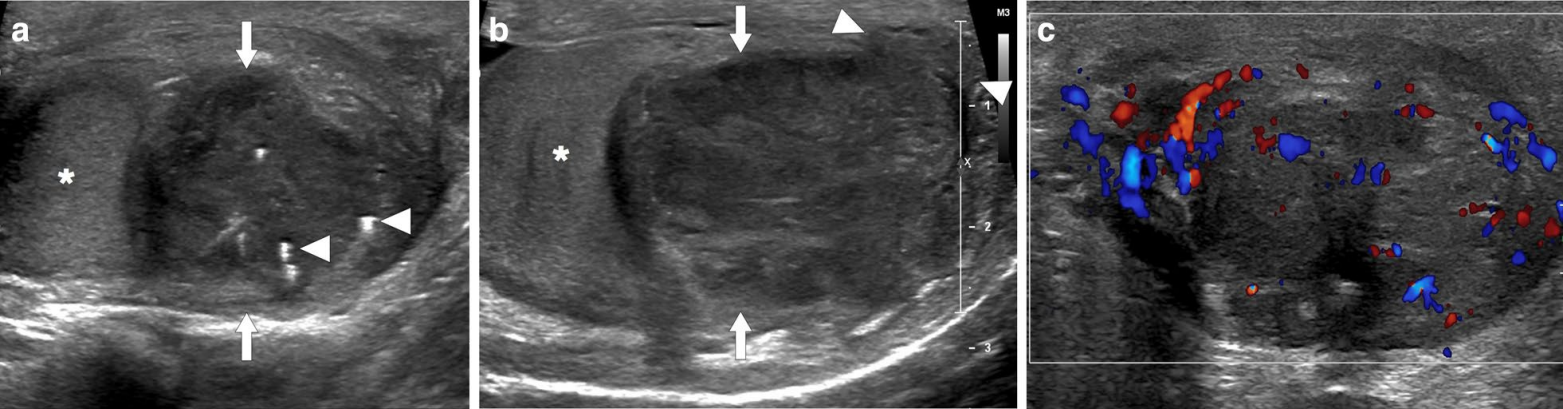
Bilateral paratesticular tuberculomas. A 63-year-old male with known metastatic renal cell carcinoma being treated with chemotherapy and bladder cancer treated with intravesical BCG therapy now presenting with bilateral scrotal masses. **a** Gray scale US image of the right hemiscrotum shows an extratesticular hypoechoic mass (arrows) adjacent to the lower pole of the right testis (asterisk). Intralesional calcifications were also noted (arrowheads). On color Doppler US, there was a mild vascularity within this lesion. **b** Gray scale US image of the left hemiscrotum shows a large heterogeneous hypoechoic mass (arrows) adjacent to the lower pole of the left testis (asterisk), compressing the testicular parenchyma and extending into the testis and scrotal wall (arrowheads). **c** Color Doppler US of the left hemiscrotal lesion reveals widespread internal vascularity. Imaging findings were considered suspicious for malignant neoplastic processes, and bilateral inguinal orchiectomy performed. Extensive inflammation with conglomerate caseating granulomas and dystrophic calcifications were seen on the pathologic specimens. Histopathological examination confirmed paratesticular tuberculoma on the right side and paratesticular, intratesticular and scrotal tuberculomas on the left side

In one case in the literature, tuberculomas were defined as well-circumscribed hypoechoic lesions with intralesional calcifications and mild internal vascularity on US (Fig. 23) [55]. It has also been reported that paratesticular tuberculomas may invade the adjacent structures such as the scrotal wall or testicular parenchyma via direct extension from the epididymis. On MRI, high T1 and low T2 signal intensities are described for these lesions, thought to be secondary to the proteinaceous content and necrotic debris [55]. Paratesticular tuberculomas may mimic malignant masses both clinically and radiologically, and histopathological examination is required for the differentiation. The presence of granulomatous inflammation with caseating necrosis and accompanying dystrophic calcifications are characteristic findings on histopathological examination.

### Polyorchidism

It is a rare congenital anomaly characterized by the presence of more than two testes. Supernumerary testes are usually located in the scrotum, less commonly in the inguinal canal or in the intraabdominal region. The most common variant is triorchidism, the presence of three testes, and the extra testis is more common on the left-side. Although most of the patients are asymptomatic, some patients may present with scrotal pain or palpable mass [56].

On ultrasound, solid nodular lesions that have similar echogenicity and echotexture to the other testes are observed [49]. On MRI, they present as solid lesions with homogeneous intermediate T1 signal intensity and high T2 signal intensity, similar to the normal testicular parenchyma [49]. They are generally smaller in size and located adjacent to the lower pole.

Additionally, bilobed testicle, also known as incomplete polyorchidism, is a very rare variant with a few cases in the literature. It has been reported that it can present as a solid nodular isoechoic lesion causing testicular contour abnormality on US, and may mimic malignancy [57].

### Splenogonadal fusion

Splenogonadal fusion is a very rare congenital anomaly arising from abnormal connection between primitive spleen and gonad during embryologic period. In the vast majority of cases, it is observed in the left testicle [49]. According to the presence of remaining connection with the spleen, two types of splenogonadal fusion have been defined, continuous and discontinuous [49]. In the continuous form, a long splenic parenchymal or fibrous cord extending from the spleen to the testis is expected and can be diagnosed with a careful sonographic examination. The discontinuous form presents as an isolated nodular lesion in the testicular parenchyma and may cause diagnostic challenges.

Splenogonadal fusion is commonly associated with inguinal hernia and cryptorchidism. Although it is generally detected incidentally, it may present as a palpable testicular mass and clinically mimic malignancy [58]. On US, it is seen as an iso-hypoechoic solid lesion with a homogeneous internal structure and internal vascularity [49, 58]. Although it is a benign condition, diagnosis before orchiectomy is difficult. In case of suspicion, Tc99m-sulfur colloid scintigraphy can help detect ectopic splenic tissue and prevent orchiectomy [49].

## Female genital tract

Female internal genitalia are a common site for neoplastic and inflammatory disease. The clinical history is of critical importance for differential diagnosis and correct diagnosis may sometimes be difficult based on imaging studies alone. A comprehensive summary of clinical and radiological findings of tumor mimickers in the female genital tract can be found in Table 3.

**Table 3:** Clinical and radiological findings of tumor mimickers in the female genital tract

Disease	Imaging findings	Auxiliary clinical information
Pelvic inflammatory disease and pelvic abscess	Pelvic fat stranding, thickening of uterosacral ligaments, fluid accumulation within the endometrial cavity, hydrosalpinx, tubal thickening, enlarged uterus and ovaries. Abscess appears as complex cystic mass with thick enhancing walls. Solid mural nodules, associated enlarged lymph nodes, peritoneal thickening and ascites may also be seen	Reproductive age. Vaginal discharge, elevated serum inflammatory markers, clinical findings of acute infection
Pelvic actinomycosis	Solid and cystic pelvic masses with diffuse spreading of the inflammatory findings across the pelvic tissue planes. Gas bubbles may be suggestive of infection	IUD is seen in most cases. Microbiological and histopathological examinations are needed for definitive diagnosis
Xanthogranulomatous oophoritis	Complex cystic lesions with thick enhancing walls and solid mural nodularity	Very rare. Surgery is needed for both diagnosis and definitive treatment
Adnexal inflammatory pseudotumors	Complex cystic adnexal masses	Rare. Histopathological examination is needed for definitive diagnosis
Endometriosis	US: Homogeneous hypoechoic cystic mass with diffuse low-level internal echoes. Internal vascularity is not expected on color Doppler US. Septation, fluid–fluid levels, thickened walls and mural nodularity related to retracting clot can be seen. May also appear as solid mass with mural/central calcifications MRI: Homogeneous, T1-hyper and T2-hypointense lesions with no enhancing solid component	Reproductive age. Pelvic pain and infertility. Catamenial complaints. Associated with increased risk of clear-cell and endometrioid type ovarian cancers
Intrauterine ovarian torsion	Complex heterogeneous cystic mass with fluid-debris levels, internal septations, calcifications and solid areas. Internal vascularity may be seen on color Doppler US	Pelvic mass in the neonatal period. Histopathological examination is needed for definitive diagnosis
Ectopic pregnancy	Complex adnexal mass with internal vascularity. Accompanying endometrial thickening due to the decidual reaction. In atypical locations, the differential diagnosis based on radiological findings may be challenging	Reproductive age. Missed menstrual period and elevated beta-hCG levels are seen mostly. Pelvic pain and vaginal bleeding. The most common locations are fallopian tubes and ovaries. History of in vitro fertilization, previous tubal surgery, pelvic inflammatory disease, IUD or congenital uterine anomalies may be suggestive
Decidualized ovarian endometrioma	US: Complex adnexal masses. Solid components generally have marked vascularity on color Doppler US MRI: Decidualized solid areas within endometriomas. Solid components have similar signal characteristics with decidual reaction in the uterine endometrium. Restricted diffusion is not expected	Pregnancy-associated disease. Previous medical history of endometriosis can be a clue. In case of suspicion, follow-up is recommended. Serial imaging shows no significant increase in size. Stable CA-125 levels may also be helpful
Hyperreactio luteinalis	Enlarged ovary with multiple ovarian cysts. Bilateral involvement is seen frequently	Pregnancy-associated disease. Mostly seen in the presence of multiple pregnancies or GTNs
Pregnancy luteoma	Solid or complex cystic ovarian lesions. Multiple lesions and bilateral involvement may be seen	Pregnancy-associated disease. Mostly detected incidentally. May present with maternal/fetal virilization. Follow-up is recommended. Generally expected to disappear in the postpartum period
Retained products of conception	US: Heterogeneous material within the endometrial cavity, thickened endometrial echo complex or intrauterine mass. Calcifications may be seen. Internal vascularity is variable MRI: Heterogeneously enhancing mass with necrotic and hemorrhagic areas	Pregnancy-associated disease. Postpartum hemorrhage and high beta-hCG levels in the early period. During follow-up beta-hCG levels are expected to return to the normal limits within 2–3 weeks

### Pelvic inflammatory disease and pelvic abscesses

Pelvic inflammatory disease (PID) is a common and prevalent infection among the reproductive age female population. Early diagnosis is critical, as there is a risk for tube-related infertility, leading to increased risk of ectopic pregnancy and chronic pelvic pain. Typical imaging features of early acute phase PID are enlarged uterus, hyperemia, parametrial fat stranding, tubal thickening, pelvic free fluid, and enlargement of the ovaries. As the disease progresses, accumulation of pus and necrotic tissue in the endometrial cavity or the fallopian tubes may ensue. The infectious material may extend to the ovaries and the peritoneum via the fallopian tubes. Pyometra, hydro- pyosalpinx, or tubo-ovarian abscess (TOA) may develop in the late phase of the disease [59].

**Fig. 24:**
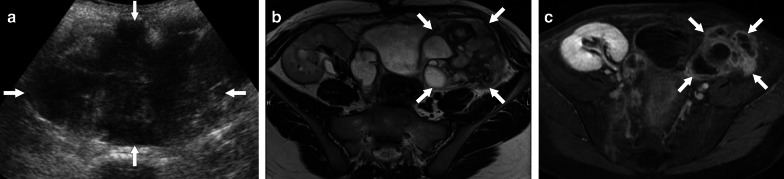
Pelvic inflammatory disease with tubo-ovarian abscess. A 23-year-old female with high fever, vaginal discharge and pain in her left lower quadrant and deep pelvis. Her medical history included chronic renal failure treated with transplantation. **a** Transabdominal sonography reveals large, complex-looking, mostly cystic left lower quadrant mass (arrows). **b** Axial T2W MR image shows a complex-looking mass with internal cystic components (arrows). **c** Axial postcontrast T1W image demonstrates intense contrast enhancement within the walls of the mass (arrows). Percutaneous aspiration produced pus, and biopsy showed intense inflammatory changes. Cultures grew *Escherichia coli*, and patient was placed on IV antibiotic treatment after aspiration

TOAs may appear as a complex cystic mass with thick enhancing walls and may also demonstrate solid mural nodules. With these imaging features epithelial ovarian tumors may be erroneously considered in these patients. The detection of vaginal discharge and elevated serum inflammatory markers are indicative for an inflammatory process. The presence of pelvic fat stranding and thickening of uterosacral ligaments are also other features of a pelvic infection. On MRI, TOAs usually have T1 hypointense fluid content with corresponding T2 hyperintensity. However, high protein content of the pus may cause elevated T1 signal on precontrast images (Fig. 24) [59, 60]. The associated enlarged lymph nodes and ascites may make the diagnosis even more challenging. The important imaging findings that favor ovarian cancer over TOA are the detection of papillary projections from the cyst wall that enhance after contrast injection, and walls or septa that measure more than 3 mm in thickness [61]. However, it is not unusual to detect large-sized pelvic abscesses which may have severely thickened contrast-enhancing septae that may be indistinguishable from epithelial ovarian neoplasms. Multiplanar primary or reconstructed images aid in diagnosis in some patients [59].

Pelvic tuberculosis is another entity that should be considered in the differential diagnosis of patients with complex adnexal masses. Tuberculous TOAs appear as multilocular lesions with necrotic components and demonstrate heterogenous enhancement on post contrast images [62].

### Actinomycosis of the female pelvis

Actinomyces species such as *A. israelii* are gram-positive anaerobic bacteria. They reside in the oral cavity, enteric system and the female genital tract. In rare cases, these organisms may cause a suppurative granulomatous process when the integrity of the mucosal barriers is breached.

In patients with pelvic actinomycosis, intrauterine device (IUD) is a common risk factor, seen in around 80% of the cases [63]. IUDs eventually get colonized by actinomyces species in 25% of the cases, and 2–4% of these colonized women ultimately develop serious actinomycotic infections [64].

**Fig. 25:**
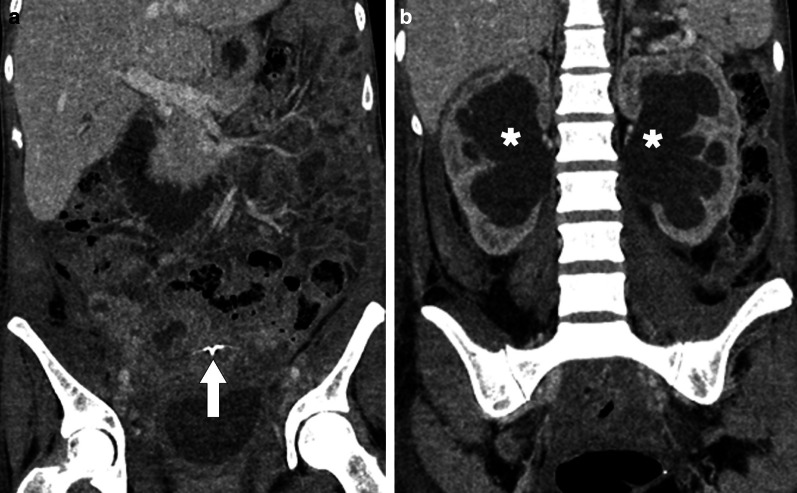
Pelvic actinomycosis. A 32-year-old woman with intrauterine contraceptive device now presenting with diffuse abdominal pain and serous vaginal discharge. **a** Coronally reformatted post contrast CT image shows the IUD (arrow). Pelvic fat planes are obliterated. **b** Coronally reformatted postcontrast CT image reveals bilateral severe hydroureteronephrosis (asterisks). Endometrial biopsy showed actinomycotic bacteria on both Gram and silver stains

Mixed solid and cystic pelvic masses may be seen in the course of the disease. A characteristic finding of pelvic actinomyces infection is the diffuse spreading of the infectious-inflammatory process across the pelvic tissue planes, due to the secretion of proteolytic enzymes that disrupt the tissue planes and boundaries (Fig. 25). The detection of gas bubbles may favor an infectious process [65]. The absence of ascites, peritoneal disease and lymphadenopathy also favor an actinomyces infection over an ovarian cancer.

### Xanthogranulomatous oophoritis

This is a very rare chronic inflammatory entity in the female genital tract manifesting with tissue destruction. Although xanthogranulomatous inflammation is generally limited to the endometrial cavity, tubo-ovarian involvement has been reported in rare cases. On imaging, the disease is seen as complex cystic lesions with thick enhancing walls and solid mural nodularity. Differentiation from neoplastic processes may be difficult based on imaging features. Surgery is mostly necessary for both confirmation of the diagnosis and definitive treatment [66].

### Adnexal inflammatory pseudotumors

**Fig. 26:**
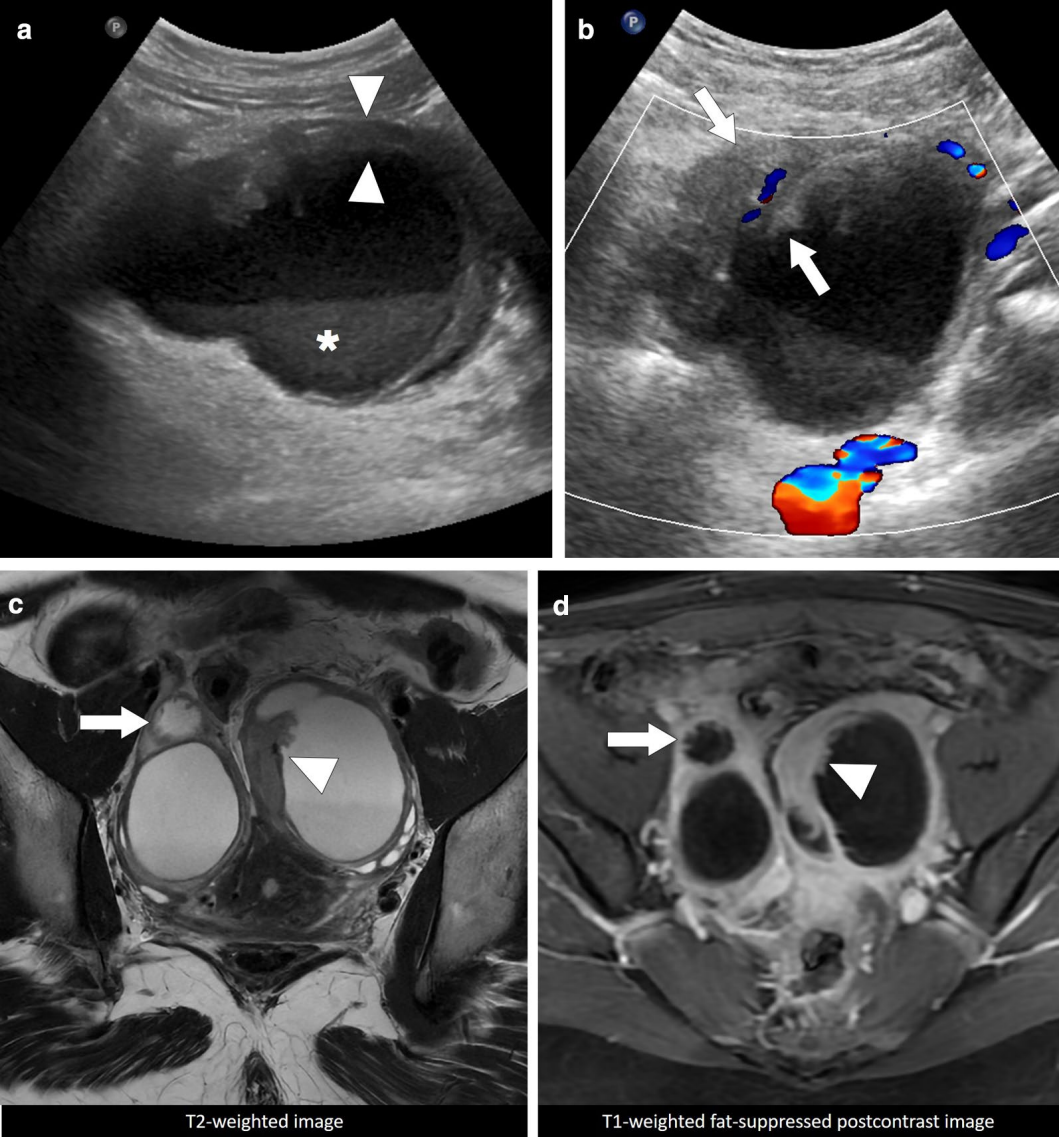
Bilateral adnexal inflammatory pseudotumor. A 25-year-old female with progressively increasing dull pain in lower abdominal quadrants which started 4–6 weeks before her initial presentation. Physical examination revealed mild abdominal tenderness and suspicious palpable masses in the pelvis without fever. Serum CA-125 level was 250 U/mL (N: 0–35 U/mL), and an ovarian malignancy was suspected based on the clinical and laboratory findings. **a** Gray scale US showed bilateral adnexal complex cystic masses with thick irregular walls (arrowheads) and layering echogenic internal debris (asterisk). **b** Color Doppler US demonstrates internal vascularity within the irregularly thickened walls (arrows). Based on sonographic findings, malignancy could not be excluded, and MRI was performed for further characterization. **c-d** Axial plane T2-weighted and T1-weighted postcontrast MR images, respectively, better demonstrated the enhancing, irregularly thickened walls of the cystic lesions in both adnexa (arrows). Histopathological examination revealed chronic inflammatory changes secondary to plasma cell and macrophage infiltration, and found consistent with inflammatory pseudotumors. Cultures from the surgical specimen did not grow any microorganism

IPTs may also be seen in the female genital system. The patients present with symptoms such as abdominal pain, fever, and weight loss. It is almost never possible to exclude malignancy in these cases solely based on imaging features as both processes appear as heterogeneous solid-cystic adnexal masses on imaging (Fig. 26). The diagnosis is usually made by histopathological examination [66].

### Endometriosis

Endometriosis is characterized by the presence of endometrial glands and stroma outside the uterus. These tissues are hormonally responsive and may cause local inflammation, bleeding, and fibrosis. It is a common cause of pelvic pain and infertility in women of reproductive age [67].

**Fig. 27:**
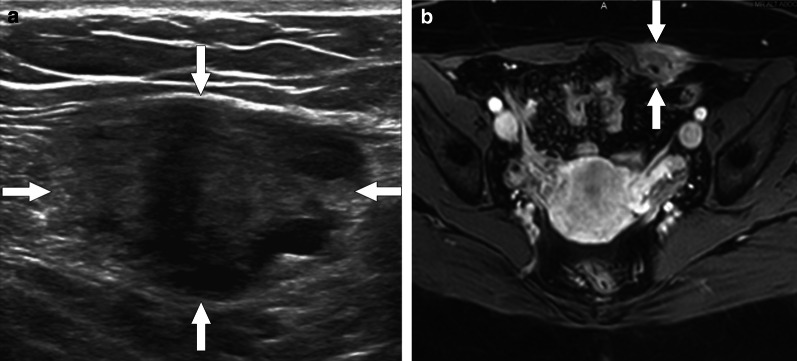
Extrapelvic endometriosis mimicking metastatic implants. A 35-year-old woman with a history of gastric adenocarcinoma now presenting with palpable mass in the abdominal wall. **a** Gray scale US image shows solid mass with lobulated contours (arrows) within the left rectus abdomens muscle. **b** Axial postcontrast fat suppressed T1W MR image shows intense enhancement of the mass (arrows). The lesion was isointense to rectus abdominis muscle on T1W image, and hypointense on T2W images with internal hyperintense foci (not shown). The lesion was resected with a preliminary diagnosis of metastatic implant but histopathologic study confirmed endometrioma within rectus abdominis muscle

Endometriosis may be asymptomatic and the clinical manifestations mostly depend on the anatomic location of the disease. Although it is generally confined to the pelvis, extrapelvic endometriosis may also occur (Fig. 27). The most common locations are ovaries, uterus, fallopian tubes, broad ligaments, uterosacral ligaments, pelvic peritoneum, pouch of Douglas, rectosigmoid colon and bladder [68].

Endometriomas may mimic malignancy from clinical and imaging standpoints. In these patients, definitive diagnosis may require histopathological evaluation. Imaging plays a crucial role for differential diagnosis and medical/surgical treatment planning [68, 69].

**Fig. 28:**
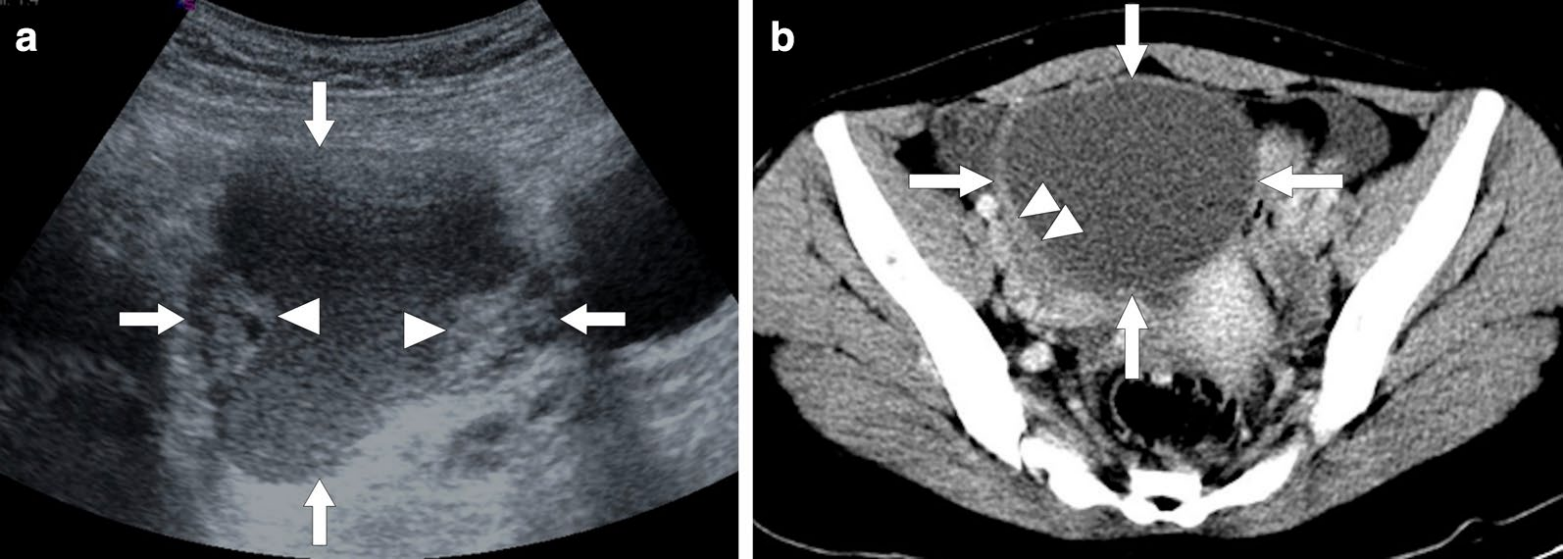
Pelvic endometriosis. A 47-year-old woman with recent onset of pelvic pain and fullness. Gray scale US image **a** showed a large cystic mass (arrows) with solid-appearing mural nodules (arrowheads). There was no apparent blood flow on color Doppler imaging. Axial plane postcontrast CT image **b** shows the same cystic lesion (arrows) with asymmetrically thickened enhancing wall (approximately 45 HU) (arrowheads). Surgical resection confirmed endometrioma with no evidence of malignant component

Morphologically endometriotic foci may range from microscopic implants to large cystic collections on a wide spectrum. The sonographic findings are highly variable and classic appearance is the presence of a homogeneous, hypoechoic cystic mass lesion within the ovary with diffuse low-level internal echoes [70]. Color flow Doppler US generally does not demonstrate internal blood flow. These ovarian endometriomas are typically referred to as “chocolate cyst” because of their thick and dark internal content due to degenerated blood products. Septations, fluid–fluid levels, thickened wall and mural nodularity due to retracting clot may be detected within these lesions (Fig. 28).

Endometriomas may sometimes appear as a completely solid mass with mural and central calcifications [67]. Differential diagnosis from ovarian neoplasia may be difficult based on imaging findings alone. The morphological complexity of these lesions may be more accentuated in patients with repeated episodes of bleeding [67, 71]. Retracted clot adhering to the lesion wall may simulate a neoplastic mural nodule which is suggestive of a malignant epithelial ovarian tumor. Adding more complexity to this situation, endometriosis has been associated with an increased risk of clear-cell type and endometrioid invasive ovarian cancers [72]. Therefore, possibility of a concomitant malignancy should also be considered in patients with endometriosis.

MRI is the preferred cross-sectional imaging modality for diagnosing endometriomas. On MRI, endometriomas generally appear as a homogeneous lesion with high signal intensity on T1W images and characteristically low signal on T2W images due to the presence of high concentrations of protein and iron [67, 68, 73]. T2 shading, a classic but non-specific MRI feature of an endometrioma, may be observed due to the cyclic hemorrhage into the cyst [74]. Subtraction images may be necessary to rule out vascularity on mural nodules that are due to retracted clot.

When evaluating endometriosis, pelvis MR imaging protocols should include T1-weighted fat-suppressed sequence as it can be quite useful in both the differential diagnosis and the evaluation of disease extent. By suppressing fat, this sequence can facilitate the differentiation of endometriomas from other T1-hyperintense adnexal lesions such as mature cystic teratomas, as well as increase the lesion conspicuity and improve the detection of smaller-sized endometriomas [73].

### Deep endometriosis

**Fig. 29:**
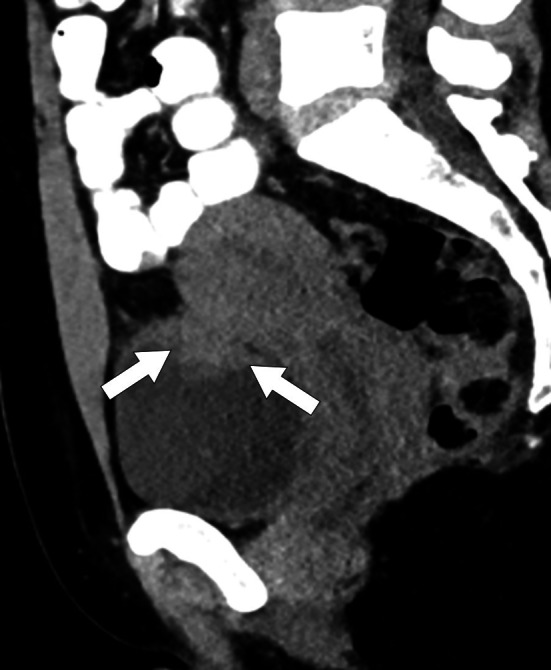
Deep endometriosis. A 40-year-old woman with no known medical history presenting with pelvic pain and hematuria. Sagittal precontrast CT image shows a nodular soft tissue mass located in the bladder dome inseparable from the anterior uterine wall. A neoplastic uterine mass invading the bladder was suspected. Surgical resection confirmed endometriosis

Deep endometriosis happens when endometrial glands and stroma infiltrate the peritoneum and pelvic ligaments or adhere to the serosal surface of the bladder or intestinal wall (Fig. 29). They may induce smooth muscle proliferation and fibrous reaction which may finally cause *solid masses*. Uterosacral ligament is the most frequent location of deep endometriosis [68].

On MRI, the foci of deep pelvic endometriosis may appear as T1 hyperintense lesions due to small hemorrhages. On T2W images hyperintense foci may be observed within the solid endometriotic masses due to the dilated ectopic endometrial glands [68].

The differential diagnosis between deep pelvic endometriosis and peritoneal implants from the gastrointestinal tract or ovarian malignancies may be difficult. The high signal intensity on T2W (most endometrial implants are T2 hypointense) is more suggestive for an implant over endometriosis. The presence of other ancillary features such as ascites and a primary tumor focus may also be helpful for diagnosing peritoneal malignant implants [68, 73, 75].

#### Chronic ovarian torsion

Ovarian torsion is among the well-known causes of acute abdominal pain. Twisting of the ovarian pedicle causing parenchymal ischemia is the underlying pathophysiological abnormality. The great majority of patients with ovarian torsion have an underlying mass which is 5 cm or larger in size [76–78]. There is a direct correlation between the size of the mass and the risk of ovarian torsion. Benign tumors are more common in these patients than their malignant counterparts [79–81]. Despite the fact that underlying ovarian mass is common in these patients, more than 50% of ovarian torsion patients who are under 15 years old have normal ovaries [80, 82]. Imaging features of ovarian torsion include the following; enlarged ovary (> 4 cm), peripherally displaced follicles with hyperechoic central stroma, free pelvic fluid and whirlpool sign of twisted vascular pedicle. The absence of venous flow on Doppler US examination is a relatively specific sign for torsion as compared to the absence of arterial flow. Ovarian echogenicity is highly variable on gray-scale US examination. It is not unusual to detect cystic spaces within the torsed ovary in patients with chronic ovarian torsion. US is typically the first modality of choice for evaluating the patients with suspected ovarian torsion. However, MRI may be more helpful in evaluating the pelvic structures in certain patients [83].

**Fig. 30:**
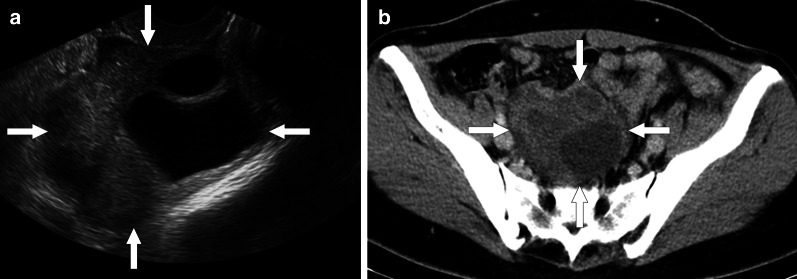
Chronic ovarian torsion. A 42-year-old female patient with no significant past medical history presenting with progressively increasing blunt pelvic pain in the last two months. **a** US study demonstrated 6 cm mostly solid appearing mass (with central and peripheral cystic areas) (arrows) within the right adnexa. Axial plane postcontrast CT scan **b** confirmed the presence of the sonographically detected mass (arrows). Postsurgical pathological examination showed no evidence of malignant disease but confirmed chronic ovarian torsion

Acute presentation is the most common form. However, the clinical course may be prolonged in the late presentation and differential diagnosis may be difficult in these patients. This clinical course is typically seen in patients with intermittent ovarian torsion which may cause delayed diagnosis [76, 84]. In these patients, torsed and edematous ovary may mimic an ovarian mass and noninvasive diagnosis may be extremely difficult if not impossible (Fig. 30).

**Fig. 31:**
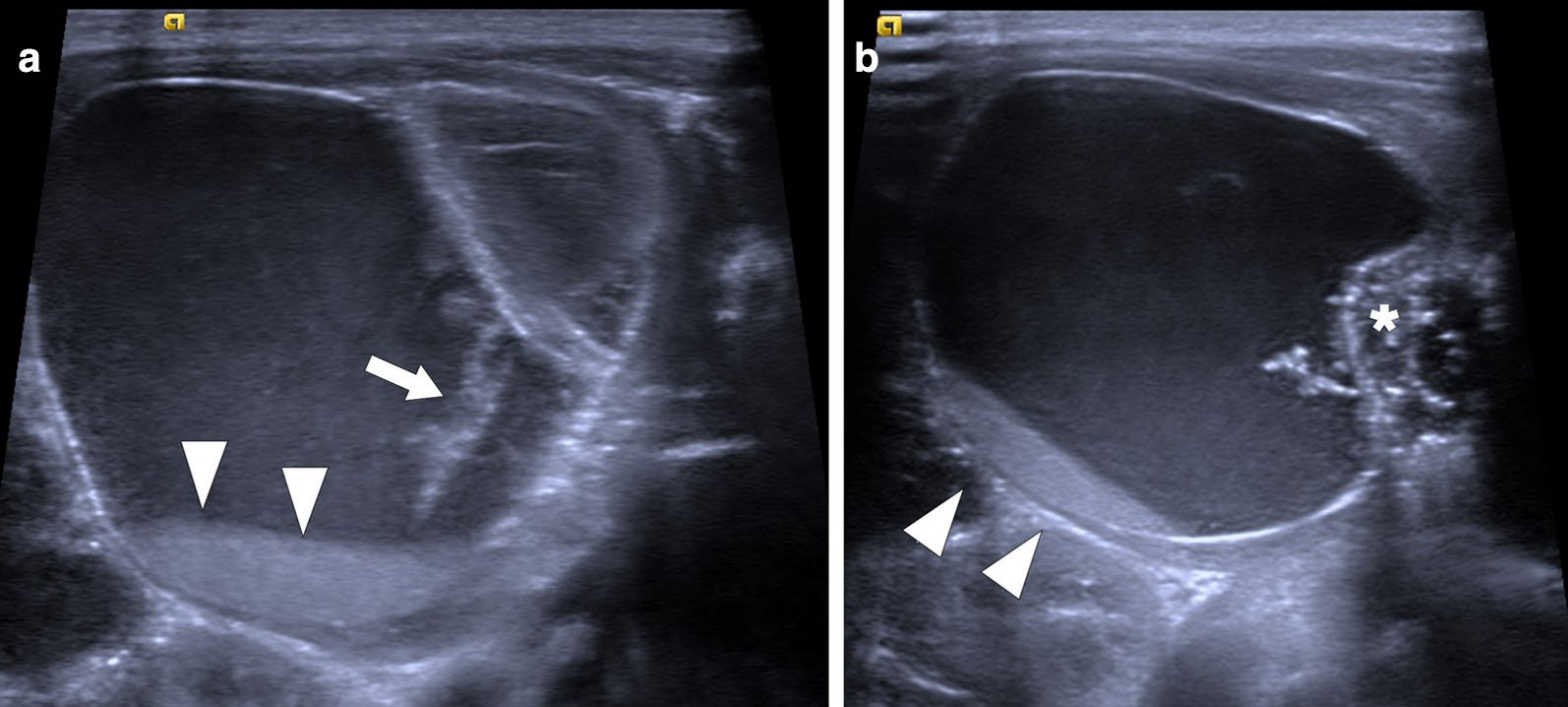
Intrauterine ovarian torsion. 10-day-old newborn girl presented with a questionable abdominal mass detected by her parents. She was otherwise asymptomatic. Physical examination was unremarkable except for palpable pelvic mass. **a**, **b** Gray scale abdominal US images showed a right adnexal complex cystic mass containing internal echogenic debris (arrowheads). The lesion was mostly cystic with irregularly thickened walls, internal septations (arrow), and a solid component with scattered calcifications (asterisk). Surgical resection and subsequent histopathological examination revealed cystic lesions with internal hemorrhage. The thick walls of the lesion were found to be non-neoplastic and composed of widespread fibrosis, necrosis and dystrophic calcifications. Findings were considered consistent with in utero ovarian torsion. Her recovery was uneventful and she was discharged 6 days after the surgery

Intrauterine ovarian torsion may present as a complex heterogeneous cystic mass in the neonatal period and may cause a diagnostic confusion by mimicking neoplastic lesions such as teratomas. On US, fluid-debris level, internal septations, retracting clot, calcifications and solid areas may all be seen (Fig. 31). Doppler US may show internal vascularity in some patients and its presence cannot confidently rule out ovarian torsion [85]. The diagnosis is mostly done after surgery. Histopathological examination may reveal autoamputation of the ovary, hemorrhagic infarcts, necrosis, dystrophic calcifications, and fibrotic tissue [85, 86].

### Ectopic pregnancy

Ectopic pregnancy (EP) is a condition characterized by the implantation of a fertilized ovum outside the endometrial cavity. The most common locations are fallopian tubes (95%) and ovaries (3%) [87]. Among the other rare locations, uterine cervix, cesarean scar or abdominal cavity may be mentioned. Basic risk factors for EP include in vitro fertilization, history of pelvic inflammatory disease, previous tubal surgery, IUD, and congenital uterine anomalies [87]. Common presenting symptoms are pelvic pain and vaginal bleeding. Acute rupture may be life threatening.

The presence of missed menstrual period with elevated serum beta-hCG levels ​​is typical for diagnosis. However, in case of chronic EP, a rare type of EP, history and beta-hCG values ​​may not be reliable. Since the number of chorionic villi is much less in chronic ectopic pregnancy, an increase in serum beta-hCG values may not be detected [88]. Moreover, the differential diagnosis of EP and gestational trophoblastic neoplasias (GTNs) may also be challenging as both pathologies can present with pelvic masses and elevated beta-hCG levels [89].

**Fig. 32:**
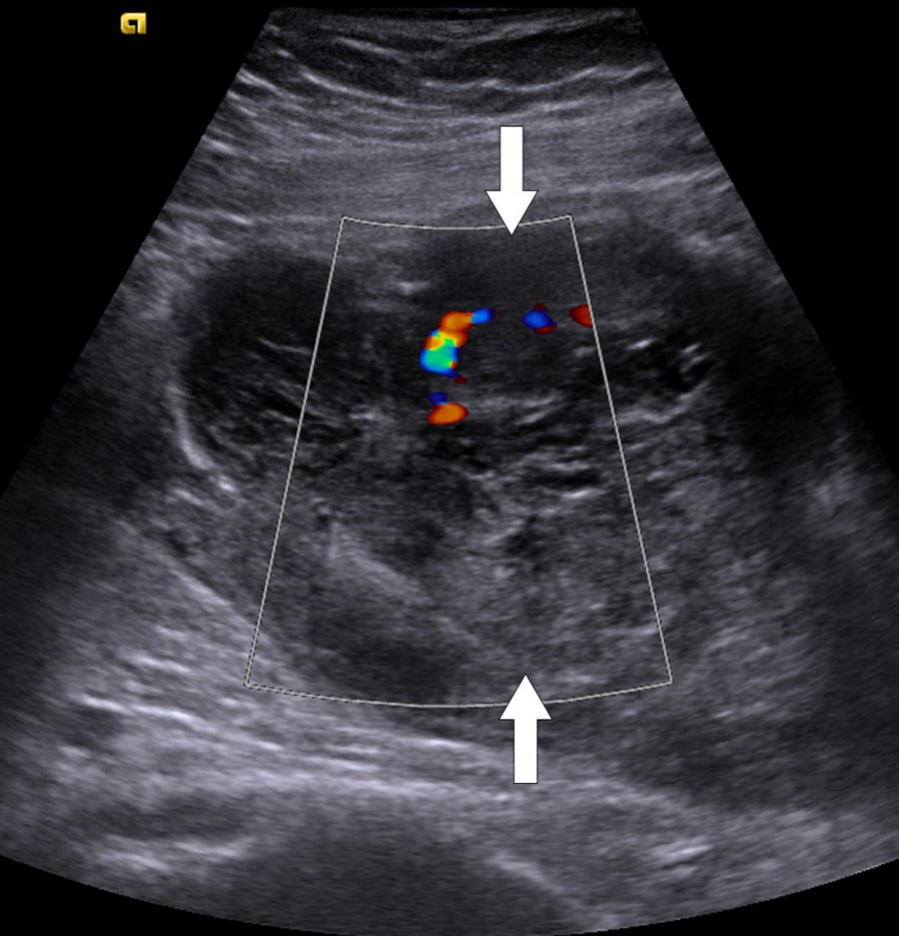
Tubal ectopic pregnancy. A 24-year-old female with abdominal pain. Gray scale transabdominal US shows a complex-appearing, mostly solid left adnexal mass with mild internal vascularization on color Doppler US examination (arrows). Serum beta-hCG level was found to be mildly elevated (1120 mIU/mL). Imaging findings suggestive of ectopic pregnancy and she was placed on methotrexate treatment. Serial follow-up US exams did not demonstrate any decrease in size and her serum beta-hCG level remained persistently elevated between 1000 and 2000 mIU/mL. The lack of response to medical treatment prompted surgical removal of the mass which finally confirmed ectopic pregnancy with no histopathological evidence of neoplastic or inflammatory disease

Transabdominal and transvaginal US is very useful for evaluation and patient follow-up of EP. However, MRI is now being increasingly used as an advanced examination method due to its high soft-tissue resolution and lack of ionizing radiation [90]. In the presence of a complex adnexal mass in fertile patients, EP should definitely be considered and investigated. The concomitant detection of thickened endometrial lining, reflecting decidual reaction, in the presence of a cystic or solid adnexal lesion, in suggestive clinical context, may be helpful for diagnosing EP (Fig. 32).

**Fig. 33:**
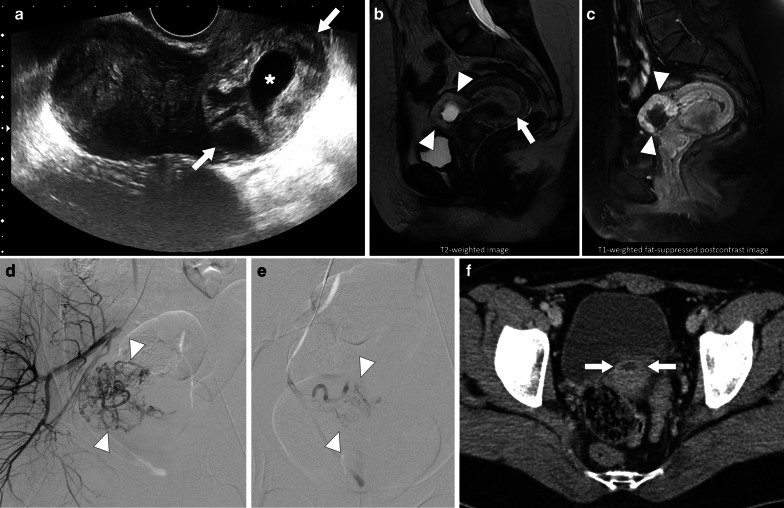
Ectopic pregnancy in the cesarean scar. A 29-year-old female patient presenting with recent onset post-coital bleeding. Gynecological examination was suspicious for a cervical mass. Serum beta-hCG level was mildly elevated (2100 mIU/mL). **a** Transvaginal gray scale US examination clearly outlined a mass (arrows) with central cystic component (asterisk) and thick walls. There were no detectable fetal elements nor fetal cardiac activity within the cystic component of the lesion. **b**, **c** Sagittal plane T2W and T1W postcontrast images, respectively, better demonstrate the same lesion with central cystic component and intense enhancement in its thick walls (arrowheads). Also note was made of thick endometrial lining suggestive of decidual reaction (arrow). The imaging and laboratory findings were inconclusive but a conservative approach was elected as the patient expressed her desire to preserve fertility. Oral methotrexate was started and a simultaneous endoarterial embolization was performed. **d** Intense vascularity was noted at the site of the lesion in pre-embolization angiography (arrowheads). **e** The lesion becomes completely devascularized after embolization (arrowheads). Her recovery was uneventful and serum beta-hCG levels precipitously dropped after these treatments. **f** Axial plane postcontrast abdominal CT image 2 year after the initial presentation showed a small residual cystic lesion (arrows), and confirmed almost complete regression of this mass

When EP develops in atypical locations such as the cervix, it is much more difficult to consider EP in differential diagnosis and it may cause diagnostic confusion [87]. However, it is important to keep in mind the possibility of an EP as it can change patient management. In suspected cases of EP, combination of more conservative approaches such as systemic methotrexate treatment, uterine artery embolization, and image-guided local methotrexate, prostaglandin, hyperosmolar glucose, NaCl or KCl administration may be preferred over surgical exploration to preserve the future fertility [91, 92]. Additionally, endovascular embolization may be a helpful precautionary intervention for gaining control of uterine arteries in case of a hemorrhagic complication (Fig. 33) [91]. In order to make an accurate diagnosis, the patient's history, patient symptoms, laboratory tests and radiological findings should be evaluated together.

### Other pregnancy-associated diseases

Adnexal masses detected during pregnancy are generally benign in nature and can be monitored conservatively with detailed clinical and radiological evaluations. The frequency of malignant lesions has been reported as less than 1% [93]. Decidualized ovarian endometrioma (DOE), hyperreactio luteinalis (HL), pregnancy luteoma (PL), and retained products of conception (RPOC) are considerable non-neoplastic entities associated with pregnancy that may mimic neoplastic processes [66].

### Decidualized ovarian endometrioma

Decidual changes expected in the endometrium during pregnancy may also rarely be seen in ovarian endometriomas and can mimic malignancy [94]. Being aware of this rare condition can enable conservative management and prevent unnecessary further interventions.

**Fig. 34:**
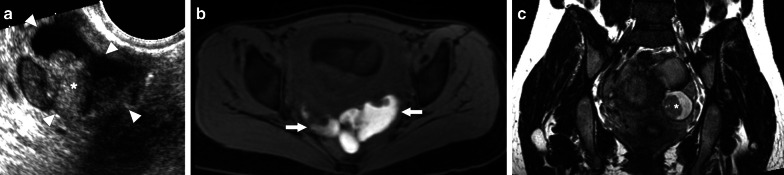
Decidualized endometriomas. A 24-year-old pregnant patient with previous history of menstrual irregularity presenting for routine obstetric US. **a** US showed a normal intrauterine pregnancy with biometry compatible with 12 weeks and 5 days gestation (not shown). However, there were bilateral (left not shown) adnexal complex cystic lesions (arrowheads) with solid components (asterisk). About 10 days later, axial T1W fat-suppressed (**b**) and coronal T2W (**c**) non-contrast pelvic MR images showed bilateral T1 hyperintense endometriomas (arrows) with T2 hypointense nodules (asterisk) in the lesions bilaterally (right not shown). She was managed expectantly. She was induced at 41 weeks and the baby was healthy. Follow-up MRI at 12 and 30 months post-partum (not shown) showed diminished size of the lesions with some complexity remaining on the left. Two years after the first delivery she delivered a second child uneventfully

On US, DOEs are seen as complex adnexal cysts containing a solid component. Marked vascularity within the solid component is a typical finding on Doppler US [94]. Since sonographic findings are mostly non-specific, MRI can be used as an auxiliary and advanced imaging modality. However, due to the concerns for teratogenic effects during pregnancy, it is usually performed without contrast. Thanks to its high soft tissue resolution, MRI better characterizes the endometriomas with their decidualized solid components that share similar signal characteristics with decidual reaction in the uterine endometrium (Fig. 34) [95]. It has also been stated that ADC values can be helpful in differentiating malignant ovarian neoplasms from DOEs. While low ADC values in the solid component were found to be associated with malignancy, high ADC values were observed in DOEs [96].

On the other hand, presence of a solid component within endometrioma should be accepted as an alarming finding in terms of malignancy, even in case of pregnancy. Therefore, even if the DOE is considered primarily, follow-up imaging should be recommended. In DOEs, significant increase in size is not expected on serial imaging [94]. Similarly, a stable course of CA-125 levels may point out benign processes [94].

### Hyperreactio luteinalis

HL is a benign condition characterized by enlarged ovaries and multiple ovarian cysts. Unilateral involvement is very unusual, and bilateral involvement is seen mostly. It is usually seen in the presence of multiple pregnancy or GTN, as it is associated with high beta-hCG levels. Rarely, HL may be seen in singleton pregnancies with normal levels of beta-hCG. Spontaneous regression during pregnancy is not expected, but it shows involution when pregnancy ends or when the underlying GTN is treated [66, 97].

### Pregnancy luteoma

PL is a rare non-neoplastic entity resulting from the excessive proliferation of luteinized stromal cells secondary to increased beta-hCG levels during pregnancy, and disappears in the postpartum period. Most of the patients are asymptomatic, and it can be detected incidentally. In 25% of the patients, endocrinological findings such as maternal or fetal virilization may be observed. While multiple lesions were reported in half of the cases, bilateral involvement was observed in one-third of the cases. They can present as solid or heterogeneous cystic lesions on imaging, and if not recognized correctly, they may result in unnecessary oophorectomy. Follow-up is recommended as their size regresses progressively, and they are generally expected to disappear within 3 months after delivery [66, 98].

### Retained products of conception

RPOC can be defined as the placental tissue remaining in the intrauterine cavity after pregnancy ends. It usually presents with postpartum hemorrhage and may be confused with GTNs due to high beta-hCG level in the early period. In particular, when RPOC coexists with placental implantation anomalies such as placenta accreta, it may be falsely interpreted as an invasive neoplastic mass [99]. During follow-up, beta-hCG levels are expected to return to the normal limits within 2–3 weeks in the presence of RPOC [100]. Persistently high beta-hCG levels, myometrial and vascular invasion, and presence of distant metastasis are findings in favor of GTNs.

On ultrasound, RPOC is seen as heterogeneous material within the endometrial cavity, thickened endometrial echo complex, or an intrauterine mass [101]. Calcifications may also be observed. On Doppler US, their vascularity varies greatly ranging from avascular lesions to the lesions with prominent internal vascularity enough to mimic artio-venous malformations [101, 102]. Signal characteristics in MRI differ according to the presence of necrosis and bleeding, and they can be seen as a heterogeneously enhancing mass that may have overlapping features with GTN [100]. Differential diagnosis of GTN and RPOC is important as it can change patient management. Conservative treatment or curettage can be applied in case of RPOC, whereas chemotherapy may be needed in GTNs [103].

## Conclusion

Genital organs are commonly affected by neoplastic and non-neoplastic diseases in both sexes. The clinical signs and symptoms are mostly non-specific and imaging is necessary for differential diagnosis. Systemic findings, clinical history, patient demographics and close communication with the referring physician are key factors, in addition to imaging findings, for accurate diagnosis. Despite all efforts, histopathological confirmation may be needed for definitive diagnosis.

## Data Availability

Data sharing is not applicable to this article as no datasets were generated or analyzed during the current study.

## Abbreviations

18-F FDG PET: Fluorine-18 fluorodeoxyglucose positron emission tomography
ADC: Apparent diffusion coefficient
CAH: Congenital adrenal hyperplasia
CT: Computed tomography
DCE: Dynamic contrast-enhanced
DOE: Decidualized ovarian endometrioma
EP: Ectopic pregnancy
GTN: Gestational trophoblastic neoplasia
HL: Hyperreactio luteinalis
IgG4-RD: Immunoglobulin G4-related disease
IPT: Inflammatory pseudotumor
IUD: Intrauterine device
LCH: Leydig's cell hyperplasia
MRI: Magnetic resonance imaging
PID: Pelvic inflammatory disease
PL: Pregnancy luteoma
PSA: Prostate-specific antigen
RPOC: Retained products of conception
STI: Segmental testicular infarction
T1W: T1-weighted
T2W: T2-weighted
TAR: Testicular adrenal rest
TB: Tuberculosis
TERT: Tubular ectasia of rete testis
TOA: Tubo-ovarian abscess
US: Ultrasonography
XGO: Xanthogranulomatous orchitis
